# High-efficiency optogenetic silencing with soma-targeted anion-conducting channelrhodopsins

**DOI:** 10.1038/s41467-018-06511-8

**Published:** 2018-10-08

**Authors:** Mathias Mahn, Lihi Gibor, Pritish Patil, Katayun Cohen-Kashi Malina, Shir Oring, Yoav Printz, Rivka Levy, Ilan Lampl, Ofer Yizhar

**Affiliations:** Department of Neurobiology, Weizmann Institute of Science, Rehovot, 7610001 Israel

## Abstract

Optogenetic silencing allows time-resolved functional interrogation of defined neuronal populations. However, the limitations of inhibitory optogenetic tools impose stringent constraints on experimental paradigms. The high light power requirement of light-driven ion pumps and their effects on intracellular ion homeostasis pose unique challenges, particularly in experiments that demand inhibition of a widespread neuronal population in vivo. *Guillardia theta* anion-conducting channelrhodopsins (GtACRs) are promising in this regard, due to their high single-channel conductance and favorable photon-ion stoichiometry. However, GtACRs show poor membrane targeting in mammalian cells, and the activity of such channels can cause transient excitation in the axon due to an excitatory chloride reversal potential in this compartment. Here, we address these problems by enhancing membrane targeting and subcellular compartmentalization of GtACRs. The resulting soma-targeted GtACRs show improved photocurrents, reduced axonal excitation and high light sensitivity, allowing highly efficient inhibition of neuronal activity in the mammalian brain.

## Abstract (web summary)

Current optogenetic inhibition methods like light-controlled ion pumps require high-intensity light and disrupt physiological ion gradients. Here, the authors somatically target the anion-conducting opsin GtACR to eliminate spiking in distal axons and improve photocurrents, thus enhancing its utility.

## Introduction

Perturbation of neuronal activity is a fundamental aspect of neuroscience research, often used to gain insight into the functional roles of particular brain regions, circuits and cell types^1^. Optogenetic tools have greatly enhanced the precision with which such manipulations can be performed^2^, providing both temporal precision and cell-type specificity to experiments aimed at defining the roles of individual neural circuit components in neural computation or animal behavior. Current optogenetic approaches for silencing of neurons are mainly based on the light-activated microbial rhodopsins halorhodopsin^3,4^, archaerhodopsin^5^, and cruxhalorhodopsin^6^. These proteins pump ions across the neuronal membrane with millisecond kinetics, independently of the electrochemical gradient, enabling neuronal silencing with precise temporal onset and offset^5,7,8^. However, ion-pumping rhodopsins possess several characteristics that impose substantial constraints on the experimental paradigm and complicate the interpretation of experimental outcomes. These limitations become even more pronounced in cases where neuronal silencing is required for extended periods of time. The unfavorable stoichiometry of one transported ion per photoreaction cycle necessitates continuous illumination at high light power. The resulting tissue heating^9,10^ and phototoxicity^11^ restrict the brain volume that is optically addressable for efficient silencing. Furthermore, ion-pumping microbial rhodopsins exhibit photocurrent amplitude decline of up to 90% after one minute of illumination, leading to reduced silencing efficacy over time^8,12,13^. Because of their insensitivity to electrochemical gradients, ion-pumping microbial rhodopsins can shift the concentrations of intracellular ions to non-physiological levels. In the case of halorhodopsin, this can lead to accumulation of chloride in the neuron, inducing changes in the reversal potential of ionotropic GABA receptors^14^. In the case of archaerhodopsin, the light-driven proton extrusion can increase the intracellular pH, inducing action potential-independent Ca^2+^ influx and elevated spontaneous vesicle release^13^. Furthermore, the hyperpolarization mediated by ion-pumping activity together with the fast off kinetics can lead to an increase in firing rate upon termination of the illumination^6,13^.

Anion-conducting channelrhodopsins (ACRs), a newly established set of optogenetic tools^15–17^, are distinct from ion-pumping rhodopsins in two major aspects: first, they can conduct multiple ions during each photoreaction cycle. This increased photocurrent yield per photon makes channelrhodopsins superior in terms of their operational light sensitivity. Second, conducting ions according to the reversal potential, ACRs are more likely to avoid non-physiological changes in ion concentration gradients. A light-gated chloride conductance can be used to effectively clamp the neuronal membrane potential to the reversal potential of chloride, which will shunt membrane depolarization, if ion permeability is sufficiently high. Anion-conducting channelrhodopsins could therefore relieve constrains imposed by ion-pumping rhodopsins. The naturally-occurring anion-conducting channelrhodopsins (nACRs) from the cryptophyte alga *Guillardia theta*^16^ are particularly interesting in this regard. These channelrhodopsins, named GtACR1 and GtACR2, have near-perfect anion selectivity and produce large photocurrents in mammalian cells, owing to a higher single-channel conductance than that of the known cation-conducting channelrhodopsins^16,18^. While GtACRs were shown to inhibit behavior in the fruit fly^19,20^ and larval zebrafish^21^, they have rarely been used in mammalian systems, most likely due to poor membrane targeting and complex effects on axonal physiology. To overcome these limitations of GtACRs and thereby of optogenetic inhibition in general, we generated several membrane-targeting-enhanced GtACR variants, converging onto soma-targeted GtACR2 (stGtACR2), a fusion construct that combines GtACR2 with a trafficking signal^22^ and a C-terminal targeting motif from the soma-localized potassium-channel Kv2.1^23^. We demonstrate here that stGtACR2 shows increased membrane targeting, high anion photocurrents and reduced axonal excitation, making it the most effective tool for optogenetic inhibition at the cell soma to date.

## Results

### GtACR2 outperforms eACRs but induces antidromic spikes

**Fig. 1:**
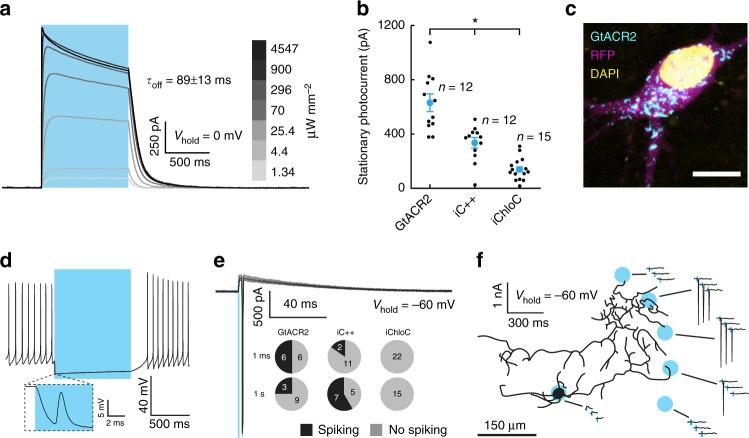
GtACR2 shows greater photocurrents than eACRs, but triggers antidromic spiking in axons. **a** Sample whole-cell voltage-clamp photocurrent recording of a GtACR2-expressing cell illuminated (470 nm) with increasing light power density. Channel closing kinetics (*τ*_off_) was determined by a single exponential fit (*I*(*t*) = *I*_0_*e^(-t*τ_off_^-1^) + *C*, *I*_0_: Current measured at light offset, *C*: holding current at *V*_hold_; mean ± SEM; *n* = 10). **b** Comparison of stationary photocurrents of blue light-sensitive ACRs (*V*_hold_ = −35 mV, current after 1 s of continuous illumination). Neurons expressing GtACR2 (*n* = 12) showed the highest photocurrents compared with neurons expressing iC + + (*n* = 12) and iChloC (*n* = 15). ANOVA, *F*(2,36) = 36.92, *p* = 1.9*10^−9^. Data are presented as mean ± SEM. **c** Representative image of GtACR2 localization. Cyan: GtACR2, magenta: cytoplasmic RFP, yellow: DAPI. Scale bar, 10 µm. **d** Representative whole-cell current-clamp recording of a GtACR2-expressing cell silenced by light application. Inset: strongly attenuated spike occurring shortly after light onset. **e** Representative whole-cell voltage-clamp recording of escaped action potentials in response to 1 ms light pulses. Pie charts depict the number of neurons with induced spikes for the three tested light-gated chloride channels: GtACR2, iC + + and iChloC. **f** Illumination of distal neurites induces spiking in cultured neurons. Schematic depicting the outline of a GtACR2-expressing neuron overlaid with the locations of laser illumination spots. Shown are whole-cell voltage-clamp responses to spatially restricted illumination at the indicated locations

To compare the utility of nACRs with eACRs for silencing of neurons, we first expressed three previously-described blue light-activated ACRs, GtACR2^16^, iC++^17^, and iChloC^15^, in cultured rat hippocampal neurons by adeno-associated virus (AAV)-mediated gene transfer. Whole-cell patch-clamp recordings from GtACR2-expressing neurons showed reliable outward photocurrents (Fig. 1a) in response to 470 nm full field light pulses. The photocurrent after 1 s of continuous illumination (stationary photocurrent) of GtACR2-expressing neurons clamped to −35 mV was significantly larger than that of the engineered ACRs (eACRs) iC++ and iChloC (628.5 ± 61.8 pA, 330.2 ± 37.9 pA, and 136.3 ± 21.4 pA, respectively; Fig. 1b). Given the poor membrane targeting and intracellular accumulation of GtACR2 (Fig. 1c), the high single-channel conductance of GtACR2^16^ is likely the cause for the high photocurrents observed in the whole-cell recordings.

We have previously shown that the red-shifted nACR GtACR1 can induce vesicle release from thalamocortical projection neurons upon illumination of their axonal terminals in the acute brain slice^13^. Malyshev and colleagues^24^ demonstrated a similar effect in GtACR2-expressing cortical pyramidal neurons in acute brain slices. Consistent with these findings, whole-cell patch-clamp recordings in current-clamp mode from GtACR2-expressing cultured neurons revealed that during a 100 ms-long illumination pulse, GtACR2 reliably inhibited action potential (AP) generation (Fig. 1d). However, this was often associated with what appeared to be an attenuated AP shortly after light onset (‘‘escaped AP’’; Fig. 1d, inset). When recorded in voltage-clamp mode, escaped APs were measured in 6 out of 12 tested GtACR2-expressing neurons in response to 1 ms light pulses at saturating light power (4.5 mW mm^−2^). These escaped spikes occurred even when the recorded photocurrent was an outward current, expected to hyperpolarize the somatic membrane (Fig. 1e, upper left). We then asked whether these light-evoked antidromic APs are specific to naturally-occurring chloride channels, or a general feature of light-evoked chloride conductance in the axon. We therefore tested two engineered anion-conducting channelrhodopsins under the same experimental conditions. APs were evoked in response to 1 ms and 1 s long light pulses in 2/13 and 7/12 iC++-expressing neurons, respectively, showing that this effect is not specific to GtACRs (Fig. 1e). No APs were evoked in iChloC expressing cells (*n* = 22 and *n* = 15, for 1 ms and 1 s long light pulses, respectively; Fig. 1e). In the case of iC++, neurons that fired APs showed a trend toward higher photocurrent amplitudes compared with those that did not (Supplementary Fig. 1). To further test the hypothesis that ACR activation might be depolarizing in some neuronal compartments, we applied spatially restricted laser pulses to the soma or neurites of cultured hippocampal neurons during whole-cell patch-clamp recordings. Light pulses directed at the soma using a galvanometric mirror system (see Methods) induced small hyperpolarizing or depolarizing photocurrents, while light pulses directed to some neurites of the same cell evoked antidromic APs (Fig. 1f).

### GtACR2-mediated antidromic spikes are reduced by KCC2

**Fig. 2:**
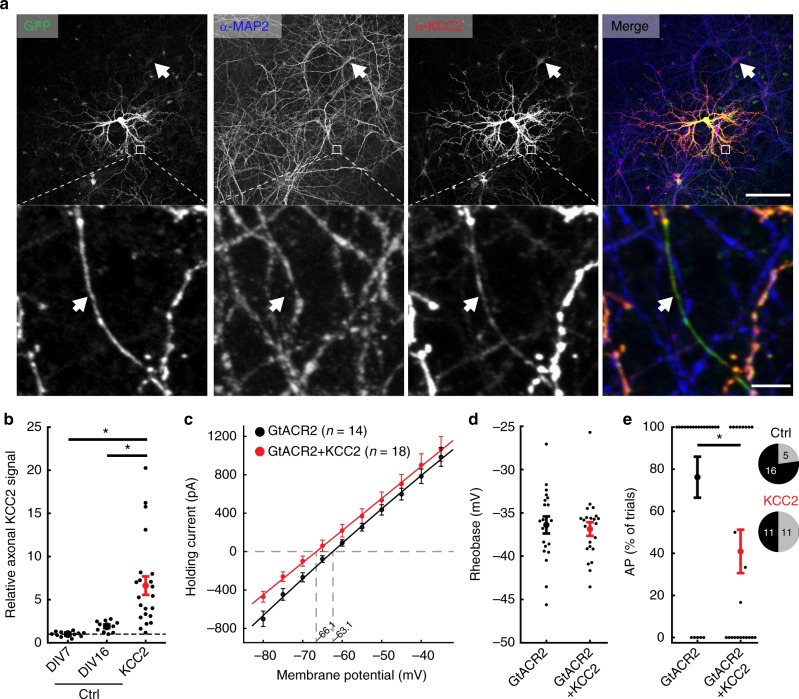
Overexpression of KCC2 reduces antidromic spiking in cultured hippocampal neurons. **a** Images from a KCC2 overexpression experiment. Endogenous KCC2 is expressed in the somatic compartment, while overexpression of KCC2 led to increased expression in axonal projections. Cultured hippocampal neurons were sparsely transfected either with GFP alone (ctrl) or GFP and KCC2. Neurons were then fixed and stained for MAP2 and KCC2. Top images show a representative region of interest with one overexpressing cell in the center. The arrow indicates a neuronal cell body expressing endogenous KCC2 levels at 16 days in vitro (DIV). Bottom images depict MAP2-expressing dendrites and a single MAP2-negative axon (arrow), which is positive for overexpressed KCC2 based on its anti-KCC2 fluorescence. Scale bars, top: 150 µm, bottom: 5 µm. **b** Quantification of axonal KCC2 immunofluorescence for immature (DIV7) and mature control neurons (DIV16) and neurons overexpressing KCC2, normalized to the average axonal KCC2 signal in immature neurons (DIV7). Axonal KCC2 fluorescence is significantly higher in KCC2-overexpressing cultures. (Kruskal–Wallis H-test, *H*(2,44) = 29.26,*p* *<* 10^−4^; ctrl: *n*_DIV7_ = 11, *n*_DIV16_ = 10, KCC2: *n* = 23) **c**–**e** Physiological properties and light-evoked spiking in cultured hippocampal neurons expressing either only GtACR2, or co-expressing KCC2. **c** Effect of KCC2 overexpression on the IV-curve. The reversal potential did not differ significantly (Student’s *t*-test, *t* = 1.5, GtACR2: *n* = 14, GtACR2 + KCC2: *n* = 18, *p* = 0.15). **d** Comparison of the minimal current injection to induce an action potential (rheobase). KCC2-overexpressing neurons did not differ from GtACR2 only-expressing neurons (Student’s *t*-test, *t* = 0.5, GtACR2: *n* = 21, GtACR2 + KCC2: *n* = 22, *p* = 0.7). **e** KCC2 overexpression significantly reduced the likelihood of GtACR2-mediated action potential generation. (Mann–Whitney, *U* = 146.5, GtACR2: *n* = 21, GtACR2 + KCC2: *n* = 22, *p* = 4*10^−2^). Seventy-six percent of GtACR2-expressing and 50% of GtACR2 + KCC2-expressing neurons showed an AP in at least one trial (pie charts). All results are presented as mean ± SEM

Based on these findings, we hypothesized that GtACR-mediated antidromic spiking results from a positively shifted chloride reversal potential in the axon, hence decreasing the axonal chloride concentration should reduce the probability of antidromic spike generation. The chloride extruder KCC2^25^ is upregulated in neurons during development, leading to high endogenous KCC2 protein levels in somatic and dendritic membranes^26^, but does not localize to axons^27–29^. We first tested whether overexpression of KCC2 leads to its localization in the axonal compartment in cultured hippocampal neurons. We co-transfected neurons with expression vectors encoding the green fluorescent protein mNeonGreen^30^ and KCC2, or with mNeonGreen alone as control. We then labeled the neurons with antibodies against the dendrite-specific microtubule-associated protein-2 (MAP2) and KCC2. Overexpression of KCC2 led to strong KCC2 immunoreactivity in dendrites and somata of transfected neurons (Fig. 2a), compared to endogenous expression levels (Fig. 2a, white arrow). To quantify axonal KCC2 levels, axons were detected as neurites that are mNeonGreen-positive and MAP2-negative (Fig. 2a, white arrows in the bottom row). While mean axonal KCC2 intensity was not significantly different between young (7 days in vitro) and mature (16 days in vitro) hippocampal cultures, KCC2 overexpression led to a 6.6 ± 1.1-fold higher axonal KCC2 signal (ctrl young vs. ctrl mature: *p* = 0.20; ctrl young vs. KCC2: *p* = 5*10^−7^; ctrl mature vs. KCC2: *p* = 9.8*10^−3^; Fig. 2b). Overexpression of KCC2 did not significantly shift the chloride reversal potential measured in the soma (Fig. 2c) or the action potential initiation threshold (rheobase, Fig. 2d), but indeed led to a significant reduction of GtACR2-evoked antidromic spiking (1 ms light pulse width, 470 nm at 4.5 mW mm^−2^; Fig. 2e). These data support the hypothesis that the chloride reversal potential in the axon is depolarizing under physiological conditions.

### Soma-targeting of GtACR2 reduces axonal excitation

**Fig. 3:**
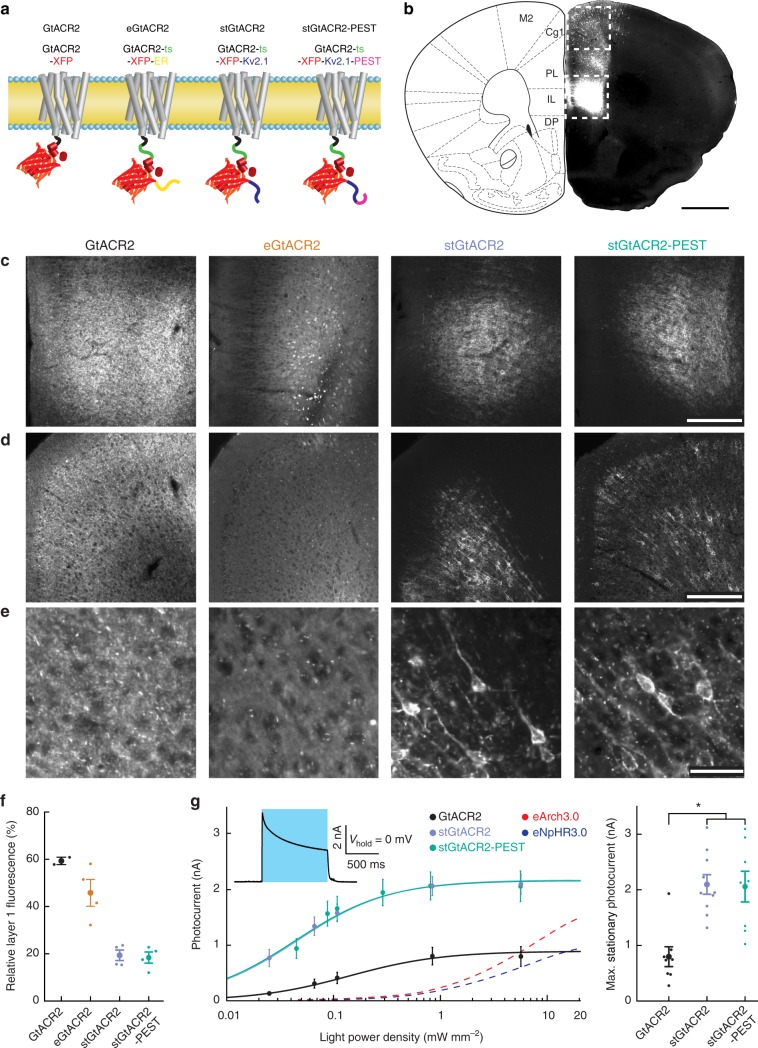
Targeting GtACR2 to the neuronal soma leads to enhanced photocurrent amplitude. **a** Schematic of different targeting approaches. GtACR2 transmembrane helix configuration based on C1C2 crystal structure^67^. **b** Image showing the fluorescence resulting from AAV-mediated cytosolic fluorophore expression in the mPFC. Transduction is most dense at the injection site (indicated by the lower dashed box) and sparse along the injection needle track (upper dashed box). Schematic adapted with permission from^68^. Scale bar, 1 mm. **c**–**e** Higher magnification images of the areas indicated in **b**. **c** Magnified images of the injection site. Scale bar, 250 µm. **d** Magnified images of the more dorsal region of sparse expression. Scale bar, 250 µm. **e** Higher magnification of **d**. stGtACR2 and stGtACR2-PEST show enrichment at the soma. Scale bar, 50 µm. **f** Quantification of soma restriction by normalizing mPFC layer 1 fluorescence by the mean fluorescence measured at the injection center. Targeting reduces relative layer 1 fluorescence (GtACR2: *n* = 2; all other groups: *n* = 4). **g** Light power density dependence of stationary photocurrents in whole-cell patch-clamp recordings of neurons in acute brain slices. Inset: Representative whole-cell voltage-clamp recording. The stationary photocurrent was defined as the photocurrent at the end of a 1 s light pulse. The photocurrent fit (*I*(LPD) = *I*_max_*LPD* (EPD50 + LPD))^−1^ was performed per cell with the effective light power density (LPD) for 50% photocurrent (EPD50) as free parameter. stGtACR2 and stGtACR2-PEST had a significantly higher maximal stationary photocurrent than GtACR2 (ANOVA, *F*(2,23) = 11.84, *p* = 2.9*10^−4^; GtACR2: *n* = 8, stGtACR2: *n* = 10, stGtACR2-PEST: *n* = 8). Fits to previously-published peak photocurrents for eArch3.0 and eNpHR3.0 are shown for reference (with permission from^8^). Results are presented as mean ± SEM

To overcome the two main caveats of GtACRs with respect to their utility as optogenetic inhibitory tools, namely, their poor membrane targeting and triggering of antidromic spikes in the axonal compartment, we designed several new GtACR2 variants with altered membrane-targeting sequences (Fig. 3a). We then tested these variants by infusing AAVs encoding them unilaterally into the medial prefrontal cortex (mPFC) of mice, leading to strong fluorescence at the injection site as well as sparse labeling along the injection needle track (Fig. 3b). Addition of the ER export and trafficking signals from the mammalian inward rectifying potassium-channel Kir2.1 was previously shown to reduce intracellular aggregation of the chloride-pump NpHR^22,31^, suggesting this approach could potentially improve membrane targeting of GtACR2. Indeed, fusion of these sequences to GtACR2 (eGtACR2) led to reduced intracellular accumulation in vivo (Fig. 3c–e). We next reasoned that removal of GtACR2 from the axonal compartment would reduce the triggering of antidromic spikes. We therefore replaced the ER export signal with the soma-targeting motif of the soma- and proximal dendrite-localized voltage-gated potassium-channel Kv2.1^32,33^, which was previously shown to enhance soma-localized expression of channelrhodopsin-2 and NpHR^23,34^. We further hypothesized that destabilizing the protein by adding a protein degradation-promoting proline (P), glutamic acid (E), serine (S), and threonine (T) rich (PEST^35^) sequence could limit the effective lifetime of membrane-resident channels diffusing along the axon, thereby further restricting GtACR2 protein levels outside the somatic compartment. The soma-targeted GtACR2, as well as the destabilized stGtACR2-PEST showed improved membrane targeting (Fig. 3c–e), strong soma-associated fluorescence and reduced neurite fluorescence (Fig. 3f). A similar expression pattern was seen in mice expressing a soma-targeted variant of the red-shifted GtACR1^16^ (stGtACR1; Supplementary Fig. 2). Functional characterization of the soma-targeted constructs by whole-cell patch-clamp recordings in the acute brain slice showed a 2.6-fold increase in stationary photocurrents compared to untargeted GtACR2, leading to average photocurrents of more than 2 nA when cells were clamped to −35 mV (Fig. 3g).

**Fig. 4:**
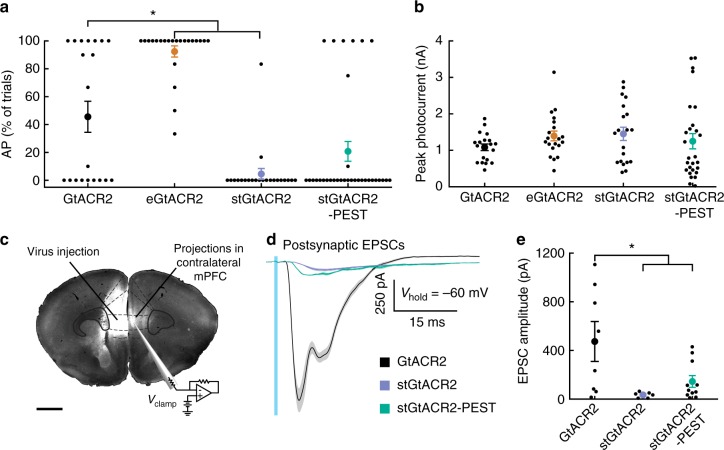
Targeting GtACR2 to the somatodendritic compartment attenuates axonal excitation. **a** Comparison of the incidence of antidromic spikes triggered by 1 ms light pulses in virally transduced cultured hippocampal neurons. eGtACR2 has a significantly increased AP incidence, while stGtACR2 decreases the occurrence of APs (Kruskal–Wallis *H*-test, *H*(3,97) = 47, *p* < 10^−4^; GtACR2: *n* = 20, eGtACR2: *n* = 22, stGtACR2: *n* = 22, stGtACR2-PEST: *n* = 33). **b** Peak photocurrents did not differ significantly between the constructs, showing that reduced AP incidence in stGtACR2 transduced neurons does not stem from smaller photocurrents (ANOVA, *F*(3,82) = 0.83, *p* = 0.48). **c** Schematic of the experimental set-up to characterize GtACR2 triggered axonal neurotransmitter release in acute brain slices. Virus encoding a cytosolic fluorophore was co-injected with the GtACR2 variants to allow for visualization of the axon terminals of transduced cortico-cortical projection neurons. Contralateral neurons in areas with high fluorescence intensity were recorded. Scale bar, 1 mm. **d** Representative traces of excitatory post-synaptic currents in response to 1 ms light pulses (470 nm, at 4.5 mW mm^−2^). **e** Quantification of the light-evoked post-synaptic current amplitude. Soma-targeting led to significant reduction in light-evoked EPSCs amplitudes (ANOVA, *F*(2,22) = 5.54, *p* = 1.13*10^−2^). All results are presented as mean ± SEM

Improved membrane targeting alone strongly increased the antidromic spike generation probability (eGtACR2; Fig. 4a), while soma-targeting not only increased photocurrents (Fig. 3g) but also decreased the probability of inducing antidromic spikes in cultured hippocampal neurons (Fig. 4a). Destabilizing stGtACR2 using the PEST sequence led to a less pronounced reduction in antidromic spike generation compared to stGtACR2 (Fig. 4a). To verify that the reduced probability of antidromic spike generation is not due to differences in peak photocurrents of the different constructs in cultured neurons, we quantified the photocurrents in the same neurons (Fig. 4b). In contrast to the stationary photocurrents in acute brain slice experiments (Fig. 3g), peak photocurrents in cultured neurons did not differ significantly between constructs, indicating a lower membrane-targeting efficiency in cultured neurons or an influence of the shorter virus incubation time. Nevertheless, it follows that the dramatic reduction in antidromic spiking for stGtACR2 is not due to lower photocurrents.

We next asked whether stGtACR2 and stGtACR2-PEST would show improved performance through reduced light-evoked synaptic release from long-range projecting axons. We injected AAVs encoding the GtACR2 variants together with a second AAV encoding a cell-filling fluorophore unilaterally to the mPFC, to allow for visualization of cortico-cortical projection neuron axons in the contralateral hemisphere (Fig. 4c). During acute brain slice preparation from these mice, the corpus callosum was severed, separating the somata of the transduced cortico-cortical projecting neurons from their axon terminals in the contralateral mPFC. Conducting whole-cell patch-clamp recordings from post-synaptic neurons in areas with fluorescently-labeled axons contralateral to the injection site therefore allowed us to characterize the isolated effect of GtACR2 activation on the axonal compartment. Blue light pulses led to reliably evoked EPSCs in slices expressing the non-targeted GtACR2 (Fig. 4d). In contrast, the EPSC amplitude was dramatically reduced in slices expressing the soma-restricted variants (Fig. 4d, e). We detected no difference in intrinsic membrane properties compared with non-expressing cells in the same acute cortical slices (Supplementary Fig. 3). The large somatic photocurrents of stGtACR2, together with the near-elimination of antidromic spiking and neurotransmitter release in distal axons, make it a highly efficient tool for optogenetic inhibition. We therefore chose to further characterize the efficacy of in vivo neuronal silencing with stGtACR2.

### Soma-targeting enhances GtACR2-mediated silencing in vivo

**Fig. 5:**
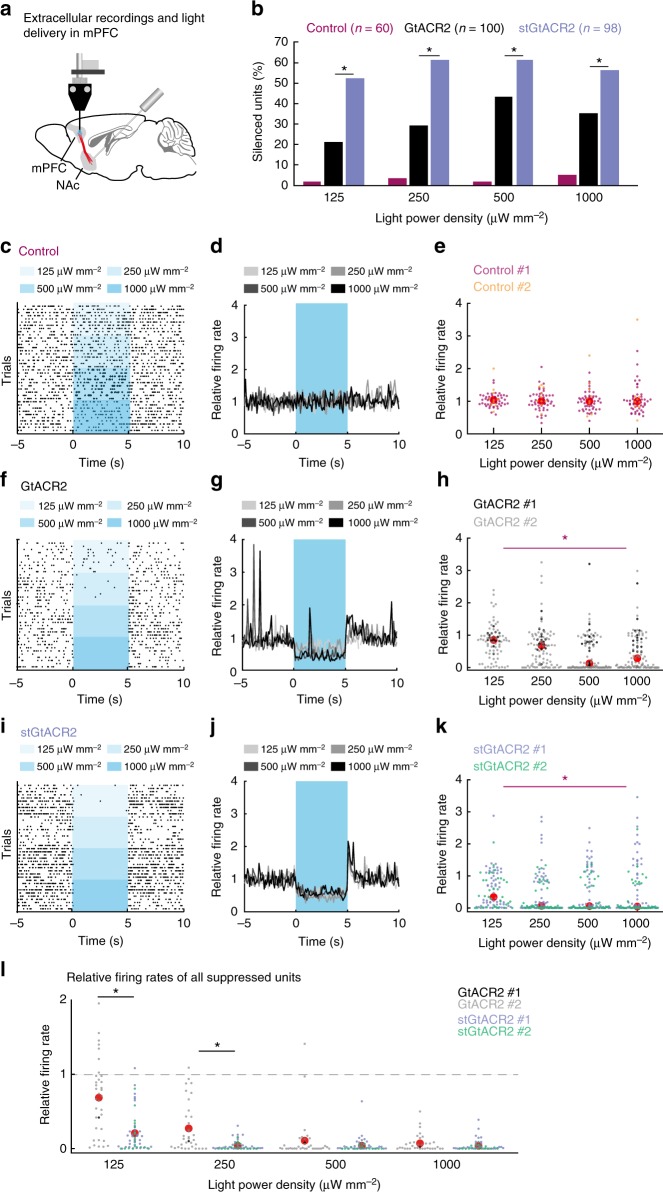
Targeting GtACR2 to the somatodendritic compartment increases its efficacy of silencing in vivo. **a** Schematic of experimental paradigm (adapted with permission from ^68^). Extracellular recordings from the mPFC were performed with a movable multi-wire optrode following AAV-mediated expression of GtACR2 or stGtACR2 in the mPFC. Inhibition was characterized in response to 5 s of mPFC illumination (460 nm). Four light power densities were tested, ten repetitions each. **b** Percentage of units with significantly reduced firing rates compared to a 5 s pre-light period. **c** A representative raster plot of a unit recorded in a non-transduced control mouse. **d** Peri-stimulus time histograms depicting the average relative firing rate (FR during light/pre-light-FR, 100 ms bins) of all recorded units. **e** Relative firing rate (FR during light/pre-light-FR) of all units recorded from non-expressing mice (*n* *=* 60 units from *n* = 2 mice in *n* *=* 10 recording sites). Each data point corresponds to a single unit, and its color corresponds to the mouse from which it was recorded. Red circles denote median values across all recorded units. **f**–**k** As **c**–**e** for GtACR2-injected mice (**f**–**h**; *n* *=* 100 units from *n* = 2 mice in *n* *=* 13 recording sites) and for stGtACR2-injected mice (**i**–**k**; *n* *=* 98 units from *n* = 2 mice in *n* *=* 13 recording sites). The response to light delivery was significantly different from control mice (repeated measures ANOVA; *p < 0.01, statistical tests and exact *p*-values are stated in the Results). Data points with values larger than 4 (GtACR2: *n* = 1; stGtACR2: *n* = 3 such points) are not shown in order to improve data visibility. Statistical tests and descriptive statistics were computed using all data. **l** Reduction in firing rate (FR during light/pre-light-FR) of units that showed significant FR suppression. stGtACR2 enables higher-efficiency neuronal suppression than GtACR2 at the two lowest light powers tested (repeated measures ANOVA; *p < 0.02, statistical tests and exact *p*-values are stated in the Results; each data point corresponds to a single unit, and its color corresponds to the mouse from which it was recorded. Red circles denote mean values across all displayed units. See Supplementary Table 1 for individual mouse data)

To examine the utility of stGtACR2 for in vivo silencing of neurons, we next characterized the efficiency of neuronal suppression by GtACR2 and stGtACR2 in awake, behaving mice (Fig. 5a). To quantify the efficiency of silencing in a large cortical volume, we performed extracellular recordings from mice expressing GtACR2 in mPFC neurons. Mice were implanted with movable fiberoptic-coupled microwire arrays in which electrodes were cut 500 µm below the optical fiber tip (Fig. 5a). Using analytical modeling of light scattering and absorption in brain tissue^36^, we estimated the light power density at the position of the extracellular recording site to be 0.11% of the light power density exiting the optical fiber (Supplementary Fig. 4). In mice expressing GtACR2 (*n* = 100 units from *n* = 2 mice in *n* = 13 recording sites), 21, 29, 43, and 35% of units significantly reduced their firing rate during 5 s pulses of blue light (460 nm) at the four light power densities tested (0.125, 0.25, 0.5 and 1 mW mm^−2^, respectively; Fig. 5b). In mice expressing stGtACR2 (*n* = 98 units from *n* = 2 mice in *n* = 13 recording sites), a greater fraction (52%, 61%, 61%, and 56%, respectively) of recorded units showed significant attenuation of firing rate during illumination with the same light power densities (Chi-square test corrected for multiple comparisons, 0.125 mW mm^−2^: *χ*^2^(1) = 20.61, *p* = 1.69*10^−5^, 0.25 mW mm^−2^: *χ*^2^(1) = 20.77, *p* = 2.07*10^−5^, 0.5 mW mm^−2^: *χ*^2^(1) = 6.59, *p* = 0.01, and 1 mW mm^−2^: *p*2(1) = 8.91, *p* = 4.96*10^−3^; Fig. 5b). In contrast, we detected no significant suppression of neuronal firing in control mice (*n* = 60 units from *n* = 2 mice in *n* = 10 recording sites) that were not injected with AAVs but similarly implanted with optrode drives (the fraction of units classified as showing a significant reduction in firing rate during illumination at any of the four light power densities was smaller or equal to the expected type 1 error rate of 5%; Fisher’s exact test: *p* = 1, *p* = 0.5, *p* = 1, *p* = 0.24, respectively). In these control mice, light at the four light powers tested had no significant effect on the firing rate of recorded units (repeated measures ANOVA, *F*(3, 168) = 0.21, *p* = 0.89; Fig. 5c–e). Single-unit recordings in mice expressing GtACR2 and stGtACR2 showed a significant effect of 5 s 460 nm light pulses on neuronal firing rates compared with controls (repeated measures ANOVA, *F*(2, 239) = 10.26, *p* = 5.3*10^−5^; ctrl vs. GtACR2: *p* = 7.17*10^−4^, ctrl vs. stGtACR2: *p* = 5.9*10^−5^; Fig. 5c–k). Units recorded from GtACR2- or stGtACR2-expressing mice also showed pronounced rebound activity upon light pulse termination (Fig. 5g, j). Comparing the magnitude of the reduction in firing rate at the different light powers revealed that stGtACR2 enables higher-efficiency neuronal suppression than GtACR2 at the two lowest light powers used (repeated measures ANOVA, *F*(3, 261) = 21.09, *p* = 2.91*10^−12^; GtACR2 vs. stGtACR2 at 0.125 mW mm^−2^: *p* = 3.2*10^−5^ and at 0.25 mW mm^−2^: *p* = 2.12*10^−4^), while inhibition was near-maximal at the two highest light powers in both cases (0.5 mW mm^−2^ and 1 mW mm^−2^, *p* = 0.90 and *p* = 1, respectively; Fig. 5l). Consistent with our slice electrophysiology recordings (Supplementary Fig. 3a, b), we found no differences in spontaneous activity in the dark between units that were effectively suppressed and those that were not in mice expressing GtACR2 or stGtACR2 (Supplementary Fig. 5; Student’s *t*-test, *p* = 0.54 and *p* = 0.45, respectively), indicating that expression of these opsins does not compromise neuronal cell health.

### Soma-targeting of GtACR2 reduces antidromic spiking in vivo

**Fig. 6:**
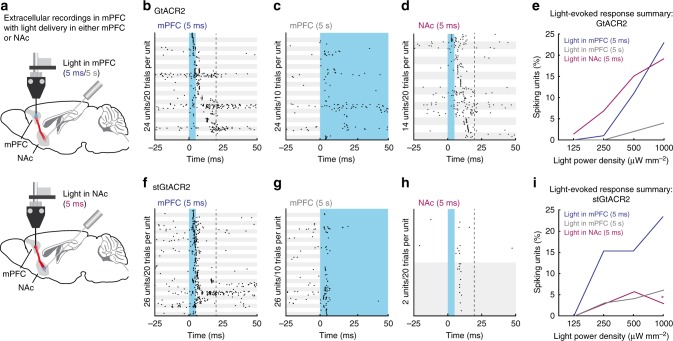
Targeting GtACR2 to the somatodendritic compartment attenuates antidromic spiking in distal axons. **a** Schematic of experimental paradigm (adapted with permission from ^68^), performed in the same mice depicted in Fig. 5. Extracellular recordings from the mPFC were performed with a movable optrode following AAV-mediated GtACR2 or stGtACR2 expression in the mPFC. Antidromic spiking was characterized in response to mPFC or NAc illumination (460 nm; mPFC − 5 ms and 5 s pulses; NAc − 5 ms pulses). Four light power densities were tested. **b**–**d** Raster plots of light onset-associated responses in units recorded from GtACR2-expressing mice. **b**, **c** All mPFC units that showed rapid light-evoked responses during a 20 ms time-window starting with the onset of either a 5 ms (**b**) or 5 s (**c**) light pulse (1 mW mm^−2^, corresponding to 28.8 mW at the fiber tip; 24 out of 100 units). Units are arranged from top to bottom according to their mean first spike latency in response to the 5 ms pulse. **d** All mPFC units that showed rapid light-evoked responses to a short (5 ms; 20 trials, 14 of 73 units) light pulse delivered to the NAc (1 mW mm^−2^). Units are arranged from top to bottom according to their mean first spike latency. **e** Summary of light-evoked responses in units recorded from GtACR2-expressing mice. Depicted are the percentages of mPFC units that showed light-evoked responses to pulses in the mPFC (5 ms, blue; 5 s, gray) and NAc (5 ms, magenta). **f**–**i** As in **b**–**e** for stGtACR2 (**f**–**g**: 26 of 98 units; **h**: 2 of 70 units). At the highest light power used, units recorded from stGtACR2-expressing mice showed a significant reduction in antidromic spiking when light was delivered to the NAc, compared with units recorded from GtACR2-expressing mice in these conditions. *p < 0.02, statistical tests and exact *p*-values are stated in the Results

Our in vitro data indicated that GtACR2 expression in the axon can lead to antidromic spiking upon illumination, and that soma-targeting of these channelrhodopsins can attenuate this effect. These recordings were performed in cultured neurons after at least 14 days in vitro, a stage at which intracellular chloride concentrations should reach the adult state^37^ as the expression of the neuronal potassium chloride co-transporter KCC2^25^ is fully upregulated^38^. Nevertheless, the chloride homeostasis of dissociated neurons may differ due to reduced concentrations of KCC2 regulators such as insulin^39^ in the culture medium. We therefore tested whether activation of GtACR2 can trigger axonal excitation in awake, behaving mice. In the same mice described in Fig. 5a, we tested whether brief light pulses delivered to the local mPFC region or to distal axons projecting to the nucleus accumbens^40^ evoke antidromic spiking (Fig. 6a). In response to brief light pulses to the mPFC (5 ms pulse width), APs were evoked (Fig. 6b–i) in the same AAV-expressing region that showed significant silencing during 5 s light pulses (Fig. 5f–l) in GtACR2- and stGtACR2-expressing mice, but not in controls. In mice expressing GtACR2, antidromic spikes were evoked in 19% of recorded units when light was delivered to the NAc (1 mW mm^−2^: 14 of 73 units; Fig. 6b–e). In mice expressing stGtACR2, in contrast, only 3% of units displayed such antidromic activity (1 mW mm^−2^: 2 of 70 units; Fig. 6f–i). These data are consistent with our slice electrophysiology results (Fig. 4), demonstrating that soma-targeting of GtACR2 significantly decreases the probability of antidromic spiking in distal axons (Chi-square test, *χ*^2^(1) = 9.58, *p* = 1.97*10^−3^). Notably, while short 5 ms light pulses to the mPFC triggered antidromic activity in both GtACR2 and stGtACR2 mice, the same units showed reduced light onset-associated spiking when measured in response to longer 5 s light pulses, consistent with shunting of the antidromic spike by the sustained chloride conductance during prolonged illumination (Fig. 6c, e, g, i). In response to 5 ms light pulses at 250 µW mm^−2^ delivered to the mPFC, more antidromic spiking units were detected in stGtACR2-expressing mice (Fig. 6f, i). This could be due to the higher membrane-targeting efficiency observed for stGtACR2 in combination with incomplete channel removal from proximal axon segments, but can be circumvented by using longer light pulses at lower light power. These data indicate that stGtACRs can serve as potent inhibitory tools for optogenetic silencing in mammalian neurons. Antidromic spiking is mostly eliminated in the distal axon by soma-targeting of GtACR2, but not in proximal axons. However, extended illumination can diminish the antidromic spiking evoked by illumination of the soma and proximal axon collaterals.

### Spatial extent of GtACR-mediated silencing

**Fig. 7:**
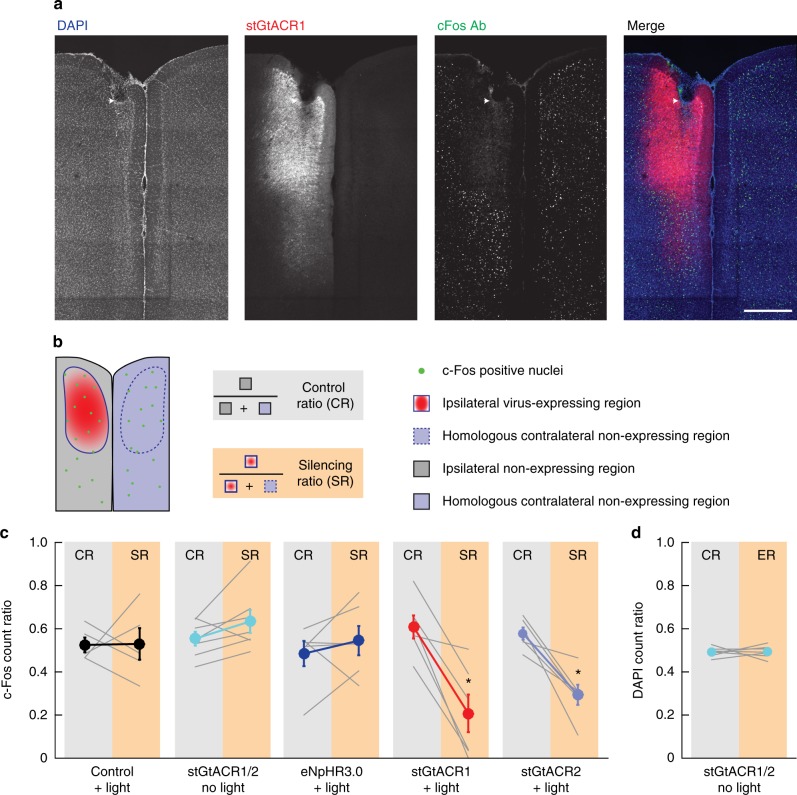
Soma-targeted GtACRs allow widespread cortical silencing. **a** Representative images of c-Fos expression in a mouse expressing stGtACR1, which was illuminated with yellow light (593 nm, 6 mW) during enriched environment exposure. c-Fos expression^69^ in the area surrounding the optic fiber implant (white arrow) was reduced. Scale bar, 500 µm. **b** Schematic of the quantification of change in the number of detected c-Fos-positive nuclei. The ipsilateral region was divided to AAV-expressing regions and regions showing no expression. The number of detected c-Fos-positive nuclei was then normalized to the sum of c-Fos-positive nuclei in a given region (i.e., either the expressing or non-expressing regions in the AAV-injected hemisphere) and its homologous contralateral non-expressing region, resulting in a ratio ranging from 0 (all detected c-Fos-positive nuclei are on the contralateral side) to 1 (all detected c-Fos-positive nuclei are on the ipsilateral side). This normalization was meant to correct for variability in the density of c-Fos-positive nuclei across different mPFC regions. If c-Fos expression is equal across hemispheres, a ratio of 0.5 is expected. **c** Measured effects under the following conditions from left to right: Light delivery (593 nm, *n* = 3 or 460 nm, *n* = 2; light at 6 mW) in fluorophore-expressing control mice (*t* = − 0.05, *n* = 5, *p* = 0.96); No light delivery in stGtACR1- (*n* = 3) and stGtACR2-expressing (*n* = 4) mice (*t* = −1.9, *n* = 7, *p* = 0.32); Illumination with 593 nm light at 6 mW in eNpHR3.0-expressing mice (*t* = − 0.88, *n* = 6, *p* = 0.84); Illumination with 593 nm light at 6 mW in stGtACR1-expressing mice (*t* = 5.2, *n* = 5, *p* = 1.78*10^−2^); Illumination with 460 nm light at 6 mW in stGtACR2-expressing mice (*t* = 4.6, *n* = 6, *p* = 2.28 × 10^−2^). Reported *p*-values are based on paired *t*-tests with Holm–Bonferroni correction. **d** Ratio of DAPI nuclei counts in non-expressing ipsilateral regions (CR) and expressing ipsilateral regions (ER) calculated as explained in **b** for c-Fos counts (stGtACR1, *n* = 3; stGtACR, *n* = 4; paired-sample *t*-test, *t* = −0.1, *n* = 7, *p* = 0.92). All results are presented as mean ± SEM

To evaluate the utility of stGtACR-mediated optogenetic inhibition in awake, behaving animals, we first compared the efficacy of these tools with that of eNpHR3.0 using c-Fos labeling. Mice were injected unilaterally in the mPFC with titer-matched AAVs encoding eNpHR3.0 (*n* = 6), stGtACR1 (*n* = 6), stGtACR2 (*n* = 6) or TagRFP (*n* = 5), and implanted with optical fibers above the site of injection. Mice were allowed to explore an enriched environment for 75 min during which continuous blue or yellow light was delivered through the optical implant (460 or 593 nm; 6 mW at the fiber tip). Immunolabeling for the immediate-early gene c-Fos (Fig. 7a) revealed a significant decrease in activity within the AAV-expressing region in mice that expressed stGtACR1 and stGtACR2, but no significant reduction was detected in mice expressing eNpHR3.0 or in control mice expressing TagRFP (Fig. 7b, c). Expression of stGtACR1 or stGtACR2 in itself did not lead to a reduction in c-Fos expression (*n* = 3 stGtACR1 and 4 stGtACR2 mice; Fig. 7c). In a recent study describing a soma-targeted GtACR1 variant^41^, high expression levels led to cell death. In contrast, expression of the stGtACR2 or stGtACR1 constructs in our study did not lead to a change in the density of DAPI-positive nuclei between the expressing and non-expressing hemispheres (Fig. 7d). These findings further indicate that stGtACR1 and stGtACR2 allow sustained inhibition of neuronal activity with high light sensitivity.

### BLA inhibition using stGtACR2 impairs fear extinction

**Fig. 8:**
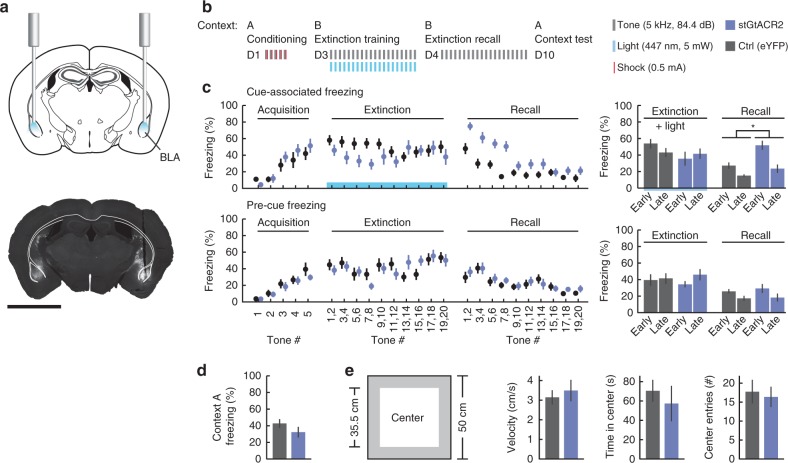
Silencing of cue-associated BLA activity using stGtACR2 suppresses extinction of cued freezing. **a** Schematic diagram depicting the location of AAV injections and fiber implants in the BLA of stGtACR2 mice (*n* = 8) and eYFP controls (*n* = 8; Schematic based on ^68^). Scale bar, 2 mm. **b** stGtACR2-expressing and control (eYFP) mice were subjected to auditory fear conditioning (day 1, conditioning), extinction training (day 3, early and late extinction) and extinction recall (day 4, early and late recall). During auditory fear conditioning mice were subjected to five tone (CS)-shock (US) presentations in context A. On day 3 twenty 30 s tone (CS) presentations were paired with light (30 s constant 447 nm light at 5 mW at the fiber tip) in context B. On day 4 extinction recall was tested by twenty 30 s tone presentations in context B. **c** Percentage of freezing during presentation of the CS (top row) and the 30 s prior to CS (bottom row). The bar graphs depict the mean percentage of freezing during the early and late phases of the trials for the two groups (early phase: first 10 CS presentations, late phase: last 10 CS presentations). Mean freezing levels for control and stGtACR2 mice, during the early recall phase, were 28 ± 4% and 54 ± 6%, respectively; the distributions of the two groups differed significantly (Scheirer Ray Hare test *H* = 4.30, *p* = 3.8*10^−2^). **d** Freezing in response to context A exposure on day 10 (Mann–Whitney *U* = 25, *p* = 0.25). **e** Characterization of the behavior of control and stGtACR2-expressing mice in an open field arena with no light delivery. No differences were found between the two groups in velocity (Mann–Whitney *U* = 29, *p* = 0.40), time spent in the center (Mann–Whitney *U* = 19, *p* = 0.09) and number of entries to the center (Mann–Whitney *U* = 31, *p* = 0.48). All results are presented as mean ± SEM

To verify that stGtACR-mediated inhibition can indeed be used to silence neurons in behavioral experiments, we applied stGtACR2 to suppress basolateral amygdala (BLA) activity during extinction of auditory-cued fear conditioning^42^. The BLA plays a central role in the acquisition as well as extinction of the conditioned freezing response. Based on previous work^43^, we hypothesized that temporally precise inhibition of the BLA during the delivery of conditioned stimuli in extinction training would suppress the formation of extinction memory. We bilaterally injected mice with AAV encoding stGtACR2 or a fluorophore-only control vector into the BLA, and implanted 200 µm-diameter optical fibers above the injection sites (Fig. 8a; Supplementary Fig. 6). Following 3 weeks of recovery, mice underwent fear conditioning in context A (Fig. 8b). Both groups (stGtACR2, *n* = 8; control, *n* = 8) showed increased freezing during acquisition (ctrl: from 3.8 ± 1.9% to 39.5 ± 8.1%; stGtACR2: from 3.5 ± 1.5% to 29.6 ± 3.5%, Scheirer Ray Hare test *H* = 52.91, *p* = 3.5*10^−10^) with no significant difference between groups (ctrl vs. stGtACR2: Scheirer Ray Hare test *H* = 0.11, *p* = 0.74), suggesting that BLA activity is not altered merely by expression of stGtACR2 (Fig. 8c). To test for fear recall and extinction, mice underwent extinction training 2 days later in a different context from that in which they were fear conditioned (context B). The extinction protocol consisted of twenty 30 s tone presentations. Each 30 s tone period was paired with constant blue light delivery (447 nm; 5mW from each fiber tip). Mice were then tested in an extinction retrieval test the following day, in which they were subjected to 20 CS presentations, but no light was delivered (Fig. 8b). During this test, stGtACR2 mice showed higher freezing rates during CS presentation (ctrl vs. stGtACR2: Scheirer Ray Hare test *H* = 4.30, *p* = 3.8*10^−2^), but freezing levels were indistinguishable from control mice during the inter-tone intervals (ctrl vs. stGtACR2: Scheirer Ray Hare test *H* = 3.6*10^−2^, *p* = 0.85; Fig. 8c), indicating that fear extinction was impaired by stGtACR2-mediated BLA inhibition during CS presentation. Sierra-Mercado and colleagues^43^ previously showed that inhibition of the BLA by muscimol injection prior to fear extinction interfered with extinction learning. Our results extend these findings, demonstrating that temporally precise inhibition of BLA activity only during CS presentation using stGtACR2 can interfere with extinction learning. Mice expressing stGtACR2 showed similar freezing levels to those of control mice during a contextual fear recall test in context A on day 10 (Fig. 8d), and similar anxiety levels measured using an open field test (Fig. 8e), indicating that stGtACR2 expression alone does not alter BLA function in fear-related behavioral paradigms. In summary, our experiments show that stGtACRs are powerful inhibitory optogenetic tools, allowing temporally precise silencing of neuronal populations in vivo.

## Discussion

We took a membrane-targeting approach to allow the utilization of the high-conductance *Guillardia theta* anion-conducting channelrhodopsins^16^ as an optogenetic tool in mammalian neurons. While these naturally-occurring channelrhodopsins show great promise due to their highly efficient photocurrents and light sensitivity^16^, and have proven effective in silencing *Drosophila* and zebrafish neurons^19–21^, they have seen limited use in mammalian neuroscience applications. This was mainly due to poor membrane targeting and complex effects on axonal excitability^13,24^. Our findings indicate that even in its non-targeted form, GtACR2 can efficiently silence neurons in the mPFC of behaving mice. These results are consistent with the high photocurrent amplitudes recorded in neurons expressing GtACR2, compared with cells expressing the engineered ACRs iC + + and iChloC^15,17^. Because of the high single-channel conductance, favorable photon-ion stoichiometry, and high light sensitivity of GtACR2, the light power density required for neuronal silencing with this opsin is at least one order of magnitude lower than that of other inhibitory opsins^8,44^. However, despite its apparent high efficacy, a significant portion of the protein seemed to reside in intracellular compartments, where it cannot contribute to functional photocurrents, and moreover carries undesirable side-effects in the form of antidromic spiking following terminal illumination. This intracellular localization is not likely to be due to GFP-fusion as similar localization was observed when GtACRs were fused to different fluorophores (not shown) and previous studies have shown little effect of fluorophore tagging of other microbial rhodopsins^45^. Furthermore, activation of GtACR2 in our recordings was also associated with antidromic spiking at light onset when illuminating both the proximal and distal axons. We have previously observed GtACR1-mediated triggering of synaptic release in thalamocortical axons^13^, consistent with recent reports of antidromic spiking in GtACR2-expressing layer 2/3 pyramidal neurons^24^ in the slice preparation. In this study, we observed GtACR2-mediated antidromic spiking in cultured hippocampal neurons, in cortico-cortical neurons in the acute slice and in cortico-striatal axons of behaving mice. Our findings indicate that axonal excitation by a chloride conductance is a general phenomenon, and could reflect a depolarized reversal potential for chloride in the axonal compartment. While such effects have been previously reported, for example in hippocampal mossy fibers^46,47^, cerebellar^48^ and brain-stem axons^49,50^, systematic evaluation of the phenomenon has been previously restricted to axons that naturally express GABA-A receptors.

To determine whether elevated chloride concentration in the axon could indeed lead to GtACR2-mediated axonal excitation, we co-expressed the KCC2 transporter with GtACR2 in cultured neurons. The endogenous KCC2 transporter, which is expressed in mature neurons and is known to be responsible for extruding chloride from the somatodendritic compartment^51^, is absent from the axon^27–29^, potentially permitting a higher chloride concentration in this compartment. Our finding that overexpression of KCC2 resulted in a significant decrease in light-induced antidromic spiking indicates that ACR-mediated antidromic spiking could indeed be the result of a smaller chloride gradient in the axon, even in adult neurons. While this antidromic spiking phenotype would probably not interfere with long-term inhibition experiments (seconds and upward), it might be a confounding factor when millisecond-scale inhibition is required. Future work could combine GtACR2 activation with red-shifted chloride indicators to directly examine changes in chloride levels in the axonal compartment during ACR-mediated chloride conductance.

Most importantly, our study demonstrates that the soma-targeted variants of GtACR2 show improved membrane expression in the somatodendritic compartment, and offer superior anion photocurrents for high-efficiency optogenetic silencing of neurons in the mammalian brain. Current optogenetic experiments often involve a sparsely labeled population of neurons that is distributed across a large brain tissue volume^52,53^. Efficient silencing of such widely-distributed neuronal populations requires continuous activity of the inhibitory optogenetic tool^54^, leading to considerable experimental constraints related to tissue heating and photodamage^9,55^. Our data indicate that stGtACRs can provide an effective means of performing such challenging experiments, due to their intrinsically high conductance, which increases their effective light sensitivity in expressing neurons. With increasing distance from the fiber tip, the wavelength-dependent transmittance becomes increasingly relevant. For instance, multiplying the action spectra of GtACR1 and GtACR2^16^ with the analytically modeled light transmittance curve^36^ for brain tissue (Supplementary Fig. 4b) reveals that excitation of GtACR2 with 480 nm or of GtACR1 with 510 nm would provide optimal light-mediated silencing at 500 µm distance from the optic fiber tip. In experiments that require optogenetic manipulation of functionally—but not anatomically—segregated neuronal populations, stGtACR2 might be combined with red-shifted tools such as C1V1^56^, Chrimson^57^ or ReaChR^58^. Red-shifted calcium sensors^59,60^ could also be used in combination with stGtACR2 due to its minimal responsivity at wavelengths above 560 nm. Notably, both stGtACR1 and stGtACR2 are also highly advantageous for multiphoton single-cell silencing experiments^45,61^ owing to their somatic restriction^23^ and high-amplitude photocurrents.

The exact parameters for neuronal silencing depend on the experimental paradigm, including the type of neuron targeted, expression level and method of light delivery. Light delivery protocols should ideally be determined by electrophysiological verification. Where transient light onset-evoked antidromic spikes are not acceptable, the utilized light power needs to be minimized. However, this leads to a restriction in the addressable brain volume when light is delivered using a classical optical fiber approach. Novel light delivery methods that reduce maximal local light power densities could help overcome this limitation (tapered fiber^62^). Rebound excitation, potentially due to the release of synaptic depression following prolonged inhibition, is a potential caveat with any optogenetic silencing experiment, and should be taken into account during experimental planning and data interpretation.

In summary, we have shown that membrane targeting and somatodendritic restriction of the naturally-occurring anion-conducting GtACR2 address two important constraints of this channelrhodopsin, greatly improving photocurrents and minimizing antidromic action potentials in distal axons. We were able to achieve high-efficiency optogenetic silencing with the optimized stGtACR variants and demonstrated their efficacy for temporally precise in vivo silencing of neuronal activity in the mouse brain.

## Methods

### Production of recombinant AAV vectors

HEK293 cells were seeded at 25–35% confluence. The cells were transfected 24 h later with plasmids encoding AAV rep, cap of AAV1 and AAV2, and a vector plasmid for the rAAV cassette expressing the relevant DNA using the PEI method^63^. Cells and medium were harvested 72 h after transfection, pelleted by centrifugation (300 × *g*), resuspended in lysis solution ([mM]: 150 NaCl, 50 Tris-HCl; pH 8.5 with NaOH) and lysed by three freeze-thaw cycles. The crude lysate was treated with 250 U benzonase (Sigma) per 1 ml of lysate at 37 °C for 1.5 h to degrade genomic and unpackaged AAV DNA before centrifugation at 3000 × *g* for 15 min to pellet cell debris. The virus particles in the supernatant (crude virus) were purified using heparin-agarose columns, eluted with soluble heparin, washed with phosphate buffered saline (PBS) and concentrated by Amicon columns. Viral suspension was aliquoted and stored at −80 °C. Viral titers were measured using real-time PCR. AAV vectors used for intracranial injections had genomic titers ranging between 8.6*10^10^ and 2*10^11^ genome copies per milliliter (gc ml^−1^). Where directly compared virus titers were matched by dilution to the lowest concentration. AAV vectors used for neuronal culture transduction were added 4 days after cell seeding. The titer was matched to final medium concentration of 1.1*10^8^ gc  ml^−1^. The AAV expression constructs described in this study are available freely on Addgene (ID 105669, 105677, 105678, and 105679).

The following viral vectors were used in this study:

AAV2/1&2.hSyn1.GtACR2-eGFP.WPRE, AAV2/1&2.CamKIIα.GtACR2-ts-Fred-Kv2.1.WPRE, AAV2/1&2.CamKIIα.GtACR2-ts-Fred-ER.WPRE, AAV2/1&2.CamKIIα.GtACR2-ts-Fred-Kv2.1-PEST.WPRE, AAV2/1&2.CamKIIα.TagRFP-T.WPRE, AAV2/1&2.CamKIIα.eYFP.WPRE, AAV2/1&2.CamKIIα.iC++-eYFP.WPRE, AAV2/1&2.hSyn.iChlOC-eGFP.WPRE.

### Primary hippocampal neuron culture

Primary cultured hippocampal neurons were prepared from male and female P0 Sprague-Dawley rat pups (Envigo). CA1 and CA3 were isolated, digested with 0.4 mg ml^−1^ papain (Worthington), and plated onto glass coverslips pre-coated with 1:30 Matrigel (Corning). Cultured neurons were maintained in a 5% CO_2_ humidified incubator with Neurobasal-A medium (Invitrogen) containing 1.25% fetal bovine serum (FBS, Biological Industries), 4% B-27 supplement (Gibco), 2 mM Glutamax (Gibco) and plated on coverslips in a 24-well plate at a density of 65,000 cells per well. To inhibit glial overgrowth, 200 µM fluorodeoxyuridine (FUDR, Sigma) was added after 4 days of in vitro culture (DIV).

### Calcium phosphate transfection of cultured neurons

Neurons were transfected using the calcium phosphate method^64^. Briefly, the medium of primary hippocampal neurons cultured in a 24-well plate was collected and replaced with 400 µl serum-free MEM medium (ThermoFisher scientific). Thirty microliters transfection mix (2 µg plasmid DNA and 250 µM CaCl_2_ in HBS at pH 7.05) were added per well. After 1 h incubation, the cells were washed two times with MEM and the medium was changed back to the collected original medium. Cultured neurons were used between 14–17 DIV for experiments.

The following plasmids were used in this study:

pAAV_hSyn1_GtACR2-eGFP_WPRE (based on a gift from Peter Hegemann, Addgene plasmid # 85463), pAAV_CamKIIα_mNeonGreen_WPRE, pAAV_CamKIIα(0.4kb)_mScarlet_WPRE, and pCITF_KCC2-tdTomato (a gift from Franck Polleux, Addgene plasmid # 61404).

### Animals

All experimental procedures were approved by the Institutional Animal Care and Use Committee at the Weizmann Institute of Science. Six-week-old C57BL/6 mice (P35–45) were obtained from Envigo. Up to 5 male or female C57BL/6 mice were housed in a cage in a light–dark (12 h–12 h) cycle with food and water ad libitum. Depending on the experiment, mice were housed for 3–12 weeks following surgery to allow for recovery and virus expression.

### Stereotactic injection of viral vectors

Six-week-old C57BL/6 mice (P35–45) were initially induced with ketamine (80 mg kg^−1^) and xylazine (10 mg kg^−1^) and placed into a stereotaxic frame (David Kopf Instruments), before isoflurane anesthesia (~1% in O2, v/v). A craniotomy (∼1 mm in diameter) was made above the injection site. Virus suspensions were slowly injected (100 nl min^–1^) using a 34 G beveled needle (Nanofil syringe, World Precision Instruments). After injection, the needle was left in place for an additional 5 min and then slowly withdrawn. The surgical procedure was either continued with optic fiber or optrode drive implantations (described below), or the surgical incision was closed with tissue glue and 0.05 mg kg^−1^ Buprenorphine was subcutaneously injected for post-surgical analgesia. Injections targeting the medial prefrontal cortex (mPFC) were made 1.8 mm anterior, 0.3 mm lateral and 2.53 mm ventral to bregma. Basolateral amygdala (BLA) injection coordinates were 1.15 mm posterior, 3.0 mm lateral, and 5.0 mm ventral to bregma. For mPFC injections, 1 µl of the indicated virus was injected. For fear extinction experiments mice were bilaterally injected with 500 nl AAV2/1&2.CamKIIα.stGtACR2-Fred.WPRE or AAV2/1&2.CamKIIα.eYFP.WPRE with a genomic titer in the range of 2–3 x 10^11^ vp ml^−1^.

### Optic fiber and optrode drive implantation

For fiberoptic implantation, a craniotomy (∼1 mm in diameter) was made above the implantation site and a ferrule-terminated optical fiber (ThorLabs) was placed at the desired coordinates using a stereotaxic frame (David Kopf Instruments). For bilateral BLA targeting, the fiber tip was placed 1.15 mm posterior, 3.0 mm lateral and 4.8 mm ventral to bregma. For nucleus accumbens, the fiber was implanted at a 45° angle with the ferrule pointing posterior to allow for optrode drive placement above the mPFC in the same animals. The fiber tip was aimed to terminate 1.42 mm anterior, 1 mm lateral and 5 mm ventral to bregma. The optical fiber was secured to the skull using Metabond (Parkell) and dental acrylic. Control mice were not injected with AAVs but implanted with an optrode drive in the mPFC and a single optical fiber in the nucleus accumbens. In mice trained for fear extinction learning additional dental acrylic was applied in a second session under isoflurane anesthesia (~1% in O_2_, v/v) after fear learning (day 2). For optrode drive implantation, the movable drive was lowered to an initial recording position above the PL (AP: 1.8 mm, ML: 0.3 mm, DV: –2.3 mm). Prior to the permanent attachment of the optrode to the skull, the optrode guide was protected with Kwik-Kast silicone elastomer (World Precision Instruments) and secured using dental acrylic. Mice were allowed to recover for at least 6 weeks before experiments. The locations of implanted optical fibers and optrodes were validated histologically for all experimental mice.

### Acute brain slice preparation

Mice were injected intraperitoneally with pentobarbital (130 mg kg^−1^, i.p.) and perfused with carbogenated (95% O_2_, 5% CO_2_) ice-cold slicing solution ([mM] 2.5 KCl, 11 glucose, 234 sucrose, 26 NaHCO3, 1.25 NaH2PO4, 10 MgSO4, 2 CaCl2; 340 mOsm). After decapitation, 300 µm coronal mPFC slices were prepared in carbogenated ice-cold slicing solution using a vibratome (Leica VT 1200 s) and allowed to recover for 20 min at 33 °C in carbogenated high-osmolarity artificial cerebrospinal fluid (high-Osm ACSF; [mM] 3.2 KCl, 11.8 glucose, 132 NaCl, 27.9 NaHCO_3_, 1.34 NaH_2_PO_4_, 1.07 MgCl_2_, 2.14 CaCl_2_; 320 mOsm) followed by 40 min incubation at 33 °C in carbogenated ACSF ([mM] 3 KCl, 11 glucose, 123 NaCl, 26 NaHCO_3_, 1.25 NaH_2_PO_4_, 1 MgCl_2_, 2 CaCl_2_; 300 mOsm). Subsequently, slices were kept at room temperature (RT) in carbogenated ACSF until use. The recording chamber was perfused with carbogenated ACSF at a rate of 2 ml min^–1^ and maintained at 32 °C.

### Electrophysiological methods for in vitro recordings

Whole-cell patch-clamp recordings were performed under visual control using oblique illumination on a two-photon laser scanning microscope (Ultima IV, Bruker) equipped with a 12 bit monochrome CCD camera (QImaging QIClick-R-F-M-12). Borosilicate glass pipettes (Sutter Instrument BF100-58-10) with resistances ranging from 3–7 MΩ were pulled using a laser micropipette puller (Sutter Instrument Model P-2000). For hippocampal neuron cultures, electrophysiological recordings from neurons were obtained in Tyrode’s medium ([mM] 150 NaCl, 4 KCl, 2 MgCl_2_, 2 CaCl_2_, 10 D-glucose, 10 HEPES; 320 mOsm; pH adjusted to 7.35 with NaOH), AcOH Tyrode’s medium ([mM] 125 NaCl, 25 AcOH, 4 KCl, 2 MgCl_2_, 2 CaCl_2_, 10 D-glucose, 10 HEPES; 320 mOsm; pH adjusted to 7.35 with NaOH). The recording chamber was perfused at 0.5 ml min^–1^ and maintained at 29°C. Pipettes were filled using standard intracellular solution ([mM] 135 K-gluconate, 4 KCl, 2 NaCl, 10 HEPES, 4 EGTA, 4 MgATP, 0.3 NaGTP; 280 mOsm kg^–1^; pH adjusted to 7.3 with KOH) or an intracellular solution allowing for EPSC and IPSC recording ([mM] 120 Cs-gluconate, 11 CsCl, 1 MgCl_2_, 1 CaCl_2_, 10 HEPES, 11 EGTA, 5 QX-314; 280 mOsm kg^–1^; pH adjusted to 7.3 with CsOH). Whole-cell voltage-clamp recordings were performed using a MultiClamp 700B amplifier, filtered at 8 kHz and digitized at 20 kHz using a Digidata 1440A digitizer (Molecular Devices).

### In vivo electrophysiology

All electrophysiological recordings in awake, freely moving mice were performed using an optrode drive consisting of an electrode bundle of 16 microwires (25 μm-diameter straightened tungsten wires; Wiretronic Inc.) attached to an 18 pin electrical connector, concentrically arranged around an optical fiber in a mechanically adjustable drive^65^. Extracellular waveform signals were collected using the Digital Lynx integrated hardware and software system (Neuralynx Inc.). The electrical signal was filtered (600–6000 Hz), amplified using a HS-18-CNR-LED unity-gain head-stage amplifier and digitized at 32 kHz. The electrode–fiber assembly was lowered by 230 or 450 µm using the mechanical drive to a new recording site at the end of each recording session, leaving at least 1 h before the next session to ensure stable recordings. Optical stimulation was applied through a ferrule-terminated optical fiber (ThorLabs) attached to the patch-cord using a zirconia sleeve (ThorLabs). For optical silencing of mPFC, we used a blue diode laser (*λ* = 460 nm, Omicron-Laserage). Light transmission for each optrode drive was measured with a calibrated power meter (ThorLabs) at the tip of the optical fiber at the end of the experiment. Light power was measured daily from the tip of the optical patch-cord before experiments. Neural data were sorted manually using Off-Line Spike Sorter 3.2.4 (OFSS, Plexon) and analyzed in Matlab (MathWorks).

### In vivo optogenetic silencing during extinction training

Mice in both the stGtACR2 and control group (eYFP expressing) were placed in the fear conditioning chamber (Med Associates) in context A. Mice were presented with five pairings of the CS (50 ms-long 5 kHz 84.4 dB tones, delivered at 10 Hz for 30 s) and US (continuous 0.5 mA foot shock for 1 s). Each CS coterminated with the US, with a 60 s interval between CS–US pairings. On day 3, mice were connected to the optical patch chord and then placed in a different chamber (context B). Context B differed from context A in the following aspects: odor (A: 1% Acetic acid vs. B: 70% EtOH), lighting (A: IR vs. B: IR + white light), box size (A: small vs. B: large), floor texture (A: grid vs. B: plain), wall texture (A: metal vs. B: Plexiglas), and background noise (A: none vs. B: fan). Mice were allowed 10 min of habituation and then presented with 20 repetitions of the CS, separated by 60 s intervals. In both groups, each 30 s CS period was paired with constant blue light (447 nm) at 5 mW, administered bilaterally from the fiber tip for the entire 30 s-duration of CS presentation. To test extinction learning, mice were placed in context B on day 4 and presented with 20 repetitions of the CS, separated by 60 s intervals. To test (with no light delivered) for potential effects of stGtACR2 expression in the BLA, fear conditioned mice were further tested in an open field test (50 x 50 cm arena lit at 160 lux) as well as for contextual freezing in context A. Movies recorded at 25 frames per second were automatically scored for freezing on day 1 and 4 by EthoVision XT 11.5 (Noldus) and by a custom written OpenCV-Python script for tracking mice that were connected to an optic fiber. The number of changed pixels compared to the last frame was quantified and filtered by a Gaussian filter with 3 frames standard deviation. When mice were connected to optical patch cords, only changed pixels around the mouse body were considered, to discard patch-cord motion. A mouse was considered to be freezing if 38 consecutive values (1.5 s) were below 983 pixels (0.5% of all pixels, EthoVision) or 100 pixels (within the ROI around the mouse, OpenCV-Python script).

### Optogenetic silencing during novel environment exploration

For all conditions except for the “no-light” condition, mice were food-deprived for 18 h and then connected to the optical patch chord and introduced to an enriched environment for 75 min Light (6 mW at the fiber tip) was continuously delivered to the mPFC during this time. The environment consisted of a circular arena (50 cm diameter) containing scattered food pellets and was dimly lit (38–44 lux) to minimize stress. Mice were then removed from the novel arena, and sacrificed after an additional 15 min. Mice in the no-light group were not implanted with optical fibers.

### Immunofluorescence and microscopy

Hippocampal neuronal cultures were fixed for 15 min with 4% paraformaldehyde in PBS. Coverslips were washed three times in PBS, incubated in blocking solution for 45 min (10% normal donkey serum (NDS) with 0.1% Triton in PBS) and then exposed overnight at 4 °C to monoclonal mouse anti-KCC2 primary antibody (diluted 1:1500 in 5% NDS, PBS; catalog # 167594 S1-12; USBiological) and rabbit anti-MAP2 (diluted 1:1000 in 5% NDS, PBS; catalog # 4542 s; Cell Signaling Technology). Following three washes in PBS, coverslips were incubated for 2 h at RT with a Cy5 Donkey Anti-Rabbit IgG (H + L) (diluted 1:500 in 5% NDS, PBS; catalog # 711-175-152; Jackson ImmunoResearch) and Cy3 Donkey Anti-Mouse IgG (H + L) (diluted 1:1000 in 5% NDS, PBS; catalog # 715-165-151; Jackson ImmunoResearch). Coverslips were then washed 2 times with PBS, dipped briefly into double-distilled water and embedded in DABCO mounting medium (Sigma). Immunostained neurons were imaged with a confocal scanning microscope (LSM 700, Carl Zeiss) using a 20x objective for overview images (NA 0.8; Carl Zeiss) and a 63x oil immersion objective (NA 1.40; Carl Zeiss) for quantification. Mice were deeply anesthetized using pentobarbital (0.4 mg g^−1^ body weight) and perfused transcardially with ice-cold phosphate buffered saline (PBS, pH 7.4) followed by a solution of 4% paraformaldehyde (PFA) in PBS. After overnight postfixation at 4 °C, brains were removed from the skull and incubated overnight in 4% PFA in PBS. Brains were stored in 30% sucrose in PBS for at least 24 h or until sectioning. Coronal sections (30 or 50 µm) were cut on a microtome (Leica Microsystems) and collected in cryoprotectant solution (25% glycerol, 30% ethylene glycol in PBS pH 6.7). For c-Fos quantification free-floating sections were washed three times in PBS, permeabilized for 1 h at RT (0.5% Triton), followed by incubated in blocking solution for 1 h (20% normal donkey serum (NDS) with 0.3% Triton in PBS) and exposed overnight at RT to polyclonal rabbit Anti-c-Fos primary antibody (diluted 1:500 in 2% NDS with 0.2% Triton in PBS; catalog # PC38-100UL; EMD Millipore). Following four washes in PBS, slices were incubated for 1 h at RT with a Cy2 or Cy5 Donkey Anti-Rabbit IgG (H + L) (diluted 1:500 and 1:200 in 5% NDS, 0.05% Triton in PBS; catalog # 711-225-152 and 711-175-152; Jackson ImmunoResearch). Sections were then washed four times with PBS and stained for 3 min with DAPI (5 mg ml^−1^ solution diluted 1:30,000 prior to staining). After three more washes in PBS, slices were embedded in DABCO mounting medium (Sigma) on gelatin-coated slides. If no staining was performed, free-floating sections were mounted on gelatin-coated slides, dehydrated, and embedded in DABCO mounting medium (Sigma). Images were acquired using a virtual slide scanner V (Olympus) or with a confocal scanning microscope (LSM 700, Carl Zeiss) using a 10x objective (NA 0.3; Carl Zeiss). Acquisition settings were kept constant within each experiment to allow for comparison between mice.

### In vitro illumination and drug application

Whole-field illumination in vitro was performed using a 470 nm light emitting diode (29 nm bandwidth LED; M470L2-C2; Thorlabs) delivered through the microscope illumination path including a custom dichroic in order to reflect the 470 nm activation wavelength. Light power densities were calculated by measuring the light transmitted through the objective using a power meter (Thorlabs PM100A with S146C sensor) and dividing by the illumination area, calculated from the microscope objective field number and magnification^66^. D-AP5 (25 µM; ab120003; Abcam) and CNQX (10 µM; C-141, Alomone) were bath applied during all culture experiments. For spatially restricted illumination of neuronal soma or neurites, a 473 nm diode laser (Bruker) was directed to the imaging plane with galvanometric mirrors, yielding a diffraction-limited spot of light that provided brief light pulses (1 ms) at each location, with 500 ms inter-pulse intervals between non-adjacent locations.

### Data analysis and statistical methods

During whole-cell patch-clamp recordings, pClamp 10 software (Molecular Devices) was used for acquisition. Data was analyzed using custom scripts written in Matlab (Mathworks). To quantify post-synaptic current amplitudes in response to light pulses, holding current traces were filtered with a Savitzky-Golay 11 point, second order, Welch window function filter and the maximal change in holding current within 20 ms (EPSCs) after light delivery was determined. Fiji (based on ImageJ2; US National Institutes of Health) was used for immunofluorescence image analysis. In the KCC2 immunofluorescence experiment all numbers (*n*) refer to the number of imaged axons. In the soma-targeting quantification and c-Fos histology, *n* refers to the number of mice. For in vitro electrophysiological recordings, *n* refers to the number of recorded neurons. For in vivo electrophysiological recordings *n* refers to the number of units, mice, or recording sites, and is explicitly stated. To detect significantly modulated units during in vivo silencing experiments, a paired-sample student’s *t*-test was performed comparing the number of detected action potentials between the 5 s pre-light period and the 5 s light period during 10 trials per light power. To detect antidromic spiking units, a paired-sample Student’s *t*-test was performed comparing the number of detected action potentials between the 20 ms pre-light period and the 20 ms light period starting with the onset of the 5 ms light pulse. Significance was determined using two tailed tests at a significance level of 0.05. For comparisons of categorical data, Chi-square test (in case no cells contained zero values) or Fisher’s exact test (in case cells contained zero values) were used. One-way analysis of variance (ANOVA) and repeated measures ANOVA were used in case of multiple comparisons where indicated. In case of non-normal data distribution and small sample sizes, non-parametric tests were used: Mann–Whitney *U*-test was used for single comparisons, the Kruskal–Wallis H-test for one-way analysis of variance, and the Scheirer Ray Hare test for two-way analysis of variance. Tukey's HSD post hoc test was used to correct for multiple comparisons. Where indicated, the *p*-value was adjusted for multiple comparisons using the Holm–Bonferroni method to correct for comparisons that are not based on the means of groups. No statistical tests were run to predetermine sample size, but sample sizes were similar to those commonly used in the field. Each experiment was performed using at least two mice or three neuronal cultures. Blinding and randomization were not performed; however automated analysis was used whenever possible.

## Electronic supplementary material

Supplementary Information

## Data Availability

The data presented in this study and custom written analysis codes are available from the corresponding authors upon request. DNA sequences are deposited on Addgene (ID 105669 and 105679).
