# Supplementary material for: High-efficiency optogenetic silencing with soma-targeted anion-conducting channelrhodopsins
Source: Nat Commun. 2018 Oct 8;9:4125. doi: 10.1038/s41467-018-06511-8 (PMC6175909; doi:10.1038/s41467-018-06511-8)
Supplement: Supplementary file 1 — Supplementary Information [file 41467_2018_6511_MOESM1_ESM.pdf]

Supplementary Information

High-efficiency optogenetic silencing with soma-targeted anion-conducting channelrhodopsins

Mahn et al. 2018

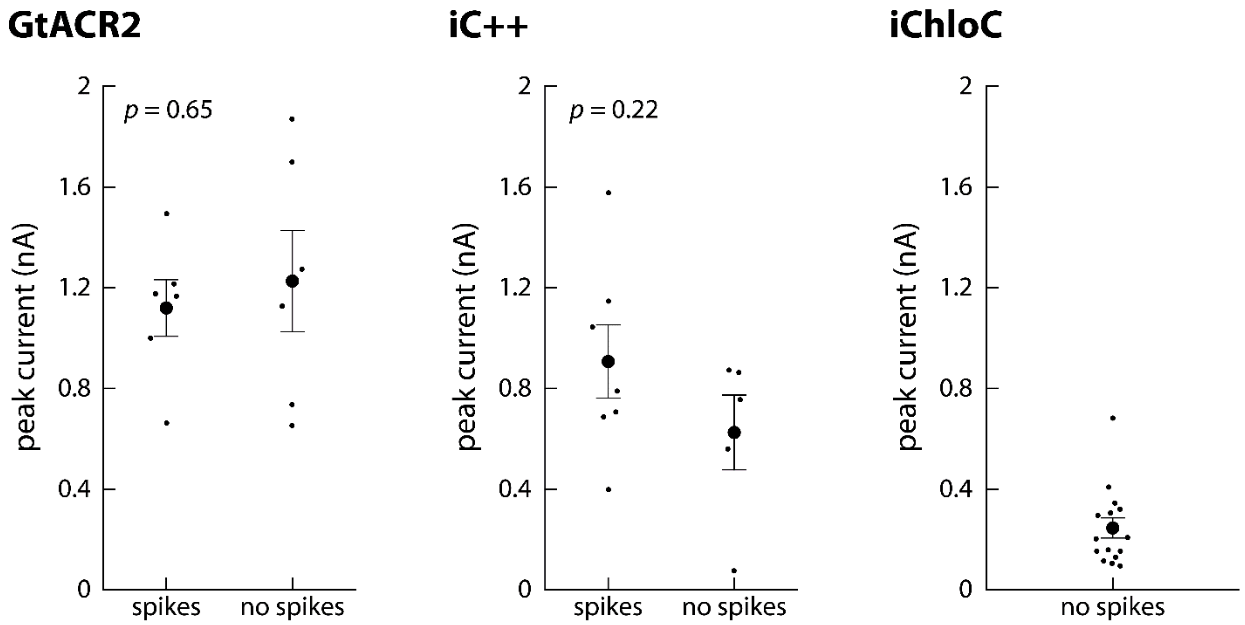

Supplementary Figure 1. Comparison of peak photocurrent amplitudes in antidromic spiking and non-spiking neurons.

Characterization of peak photocurrents in ACRs by whole-cell voltage-clamp recording from cultured rat hippocampal neurons. In each plot, photocurrent amplitudes are presented for neurons that displayed antidromic spikes (“spikes”) and those that did not (“no spikes”). None of the iChloC-expressing neurons displayed antidromic spiking. Data points from individual neurons as well as their mean ± SEM are displayed.

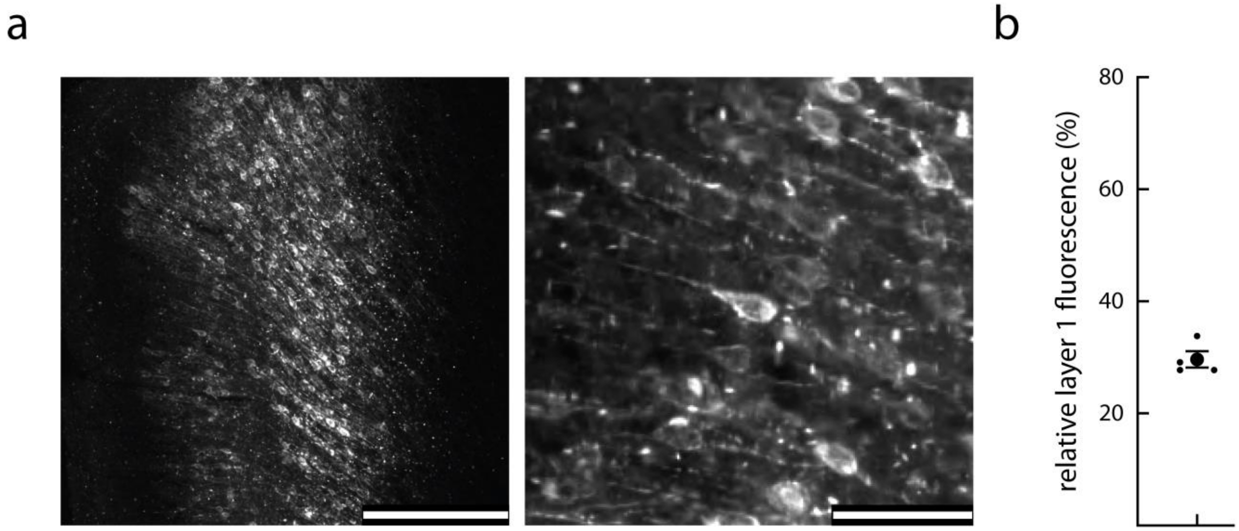

Supplementary Figure 2. Soma-targeting of the red-shifted GtACR1

(a) Confocal microscope images of coronal sections from the mPFC of mice injected with an AAV encoding stGtACR1 under the CaMKIIα promoter (pAAV-CaMKIIα-stGtACR1-FusionRed-WPRE). Scale bar on the left image: 250 µm; scale bar on the right image: 50 µm **(b)** Quantification of soma restriction of stGtACR1, calculated as in Figure 3 by normalizing mPFC layer 1 fluorescence by the mean fluorescence measured at the injection center. Data points from individual mice (*n* = 4) as well as their mean ± SEM are displayed.

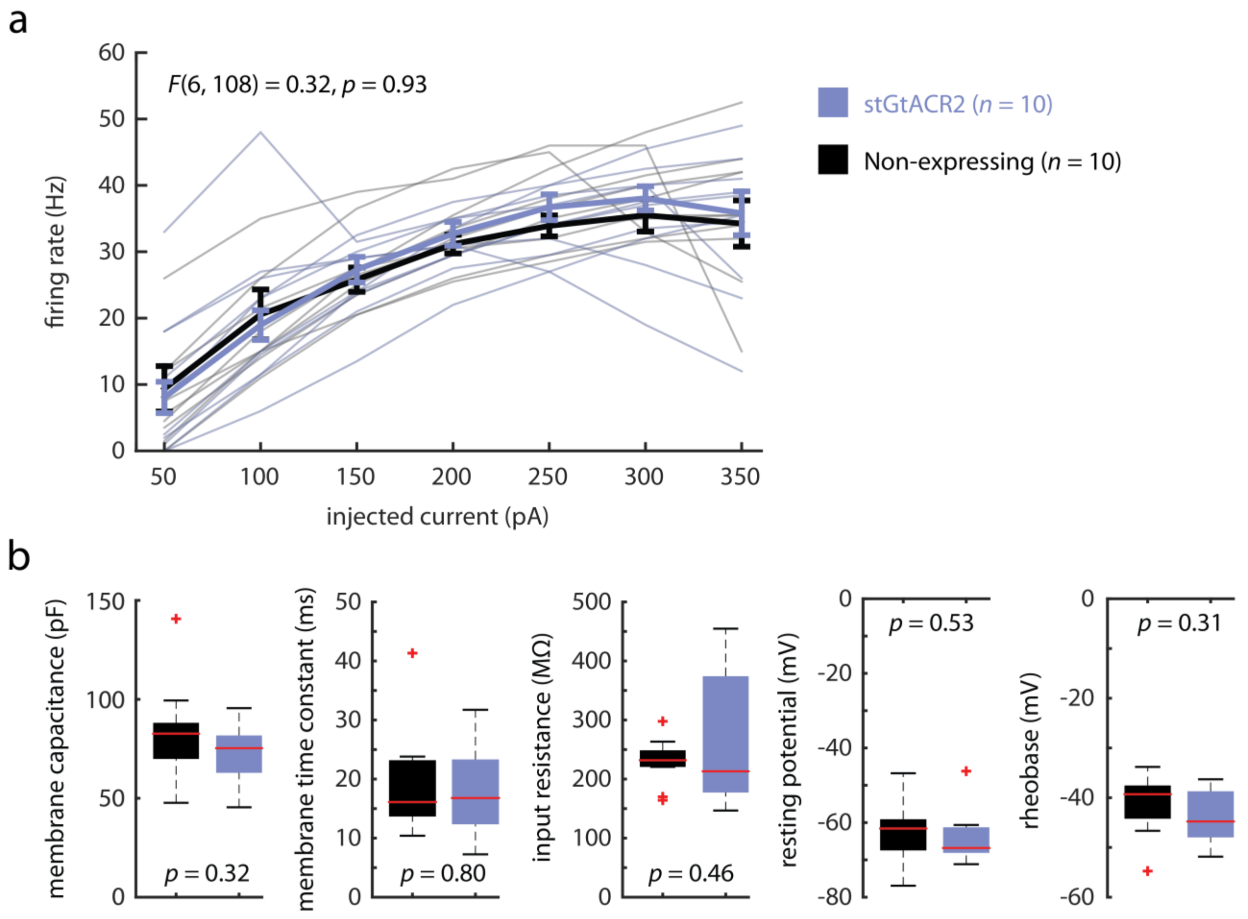

Supplementary Figure 3. Intrinsic properties of stGtACR2-expressing and non-expressing neurons in acute mPFC slices.

(a) Summary of current clamp recordings from neurons expressing stGtACR2 (n = 10) and neighboring non-expressing neurons (n = 10). Firing rates are plotted against injected current, individual cells are depicted by thin lines, while bold lines depict mean ± SEM (repeated measures ANOVA, firing rate × stGtACR2 expression interaction shown on the plot). **(b)** Comparison of intrinsic membrane properties in stGtACR2-expressing and control non-expressing neurons. Boxes depict 25th and 75th percentiles, while whiskers show 5th and 95th percentiles. Outliers are shown as red crosses. None of the comparisons yielded significant differences (unpaired Student's t-tests; p values shown on plots), indicating that stGtACR2 expression does not alter the intrinsic properties of expressing neurons.

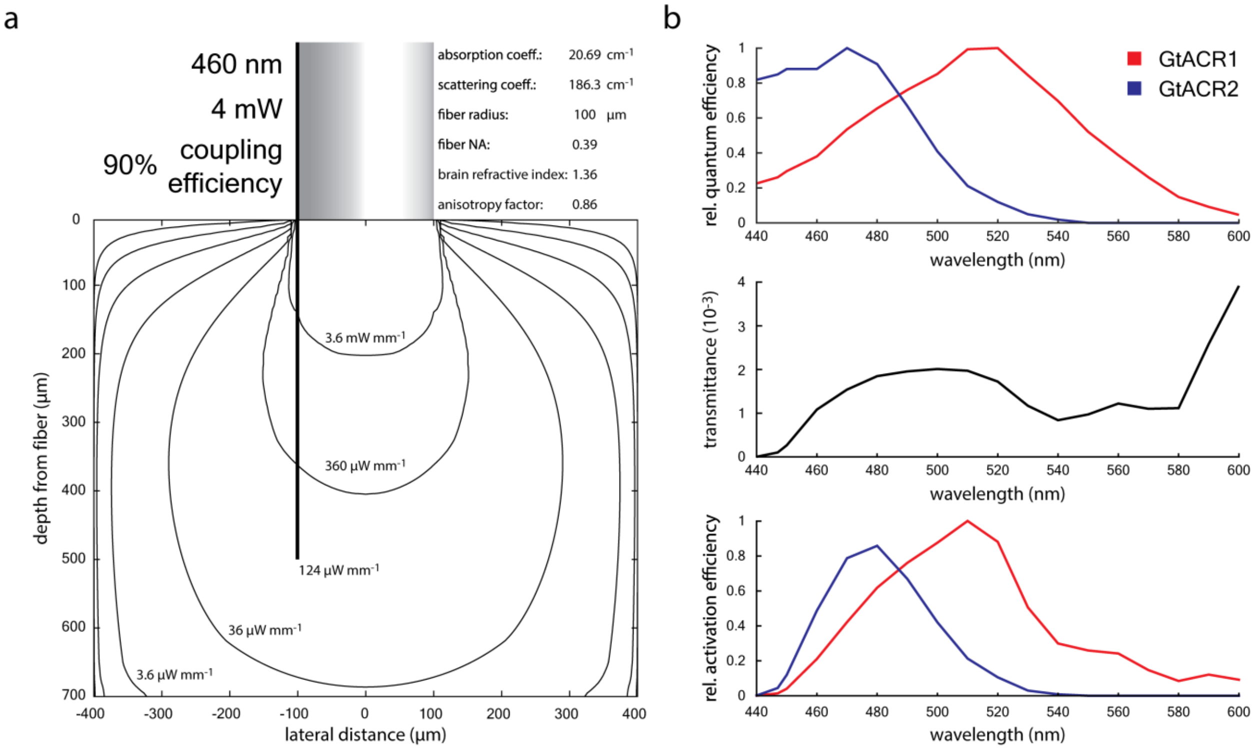

Supplementary Figure 4. Functional action spectra of GtACR1 and GtACR2 based on multiplication with analytical light spread estimation

Light propagation from a flat-cleaved optical fiber within brain tissue was estimated by analytical modeling [1], using gray matter parameters as estimated by Liu et al. [2]: Wavelength dependent brain scattering coefficiecient: $\mu_s(\lambda) = (2.37*(\lambda/500nm)-1.15) * (1-g)^{-1}$. Absorption is estimated as: $\mu_a(\lambda) = B*S*\mu_a(HbO_2(\lambda)) + B*(1-S)*\mu_a(Hb(\lambda)) + W*\mu_a(H_2O(\lambda))$. Blood oxygen saturation: S (62%). Estimated percentage of water in brain tissue: W (65%). Percentage of blood in the brain tissue (B). Cerebral cortex (PFC) blood volume excluding major vessels was estimated as 4.6% according to Chugh et al. (2009) [3]. Wavelength dependent blood and water absorption coefficients from omlc.org were used. **(a)** Contour lines of estimated light power densities resulting from 4 mW light coupled to an optical fiber. At the recording site (500 µm below and 100 µm lateral to the optical fiber center) the light power density drops to ~0.11% of the light power density at the fiber surface. **(b)** Comparison of GtACR1 and GtACR2 activation efficiency within brain tissue by including the wavelength dependent transmittance. Top: relative quantum efficiency as reported in Govorunova et al. (2015) [4]. Middle: Wavelength dependent transmittance at electrode recording site, modeled as in a. Bottom: Relative activation efficiency normalized by maximal activation of GtACR1. The higher scattering and absorption at 470 nm shift the most efficient excitation wavelength to 480 nm for GtACR2. The higher blood absorption coefficient at 520 nm results in 510 nm being the most efficient wavelength for GtACR1 activation at this distance from the optic fiber. According to this estimation, GtACR1 used at 510 nm allows for 14% lower light powers compared to GtACR2 excited at 480 nm, making GtACR1 the more efficient tool when only a single wavelength is needed. However, GtACR1 will cause non-permissive activation in the lower as well as the higher wavelength ranges, therefore only GtACR2 allows for the combination with other currently available optogenetic actuators or reporters.

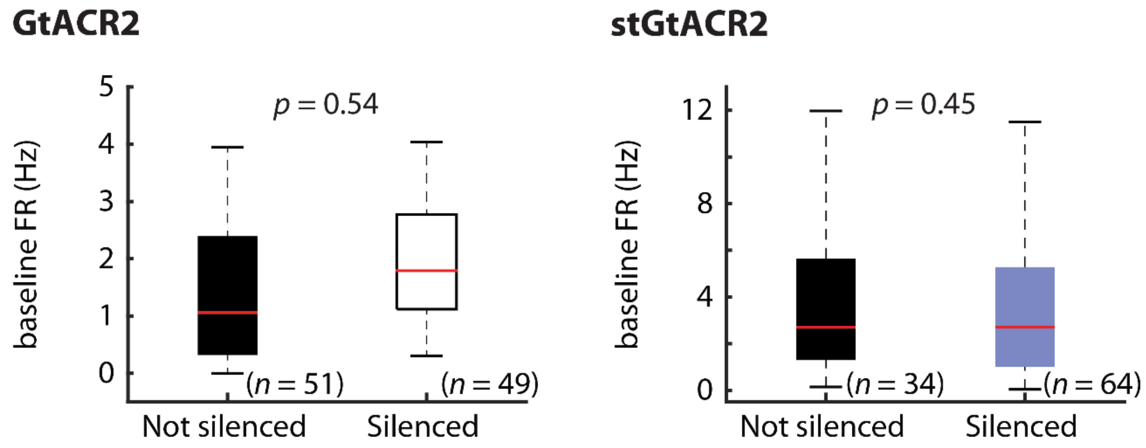

Supplementary Figure 5. Spontaneous firing rates of single units recorded in mice expressing GtACR2 and stGtACR2

Box plots depict the comparison between spontaneous firing rates of single units that showed a significant reduction in firing rates ("Silenced") during 5 s light pulses, and those of units that did not ("Not silenced"). Outliers, defined as data points that lie beyond the whiskers (length equals to 1.5 * inter-quantile range), are not shown. No difference was detected between these two populations in mice expressing either GtACR2 (left) or stGtACR2 (right).

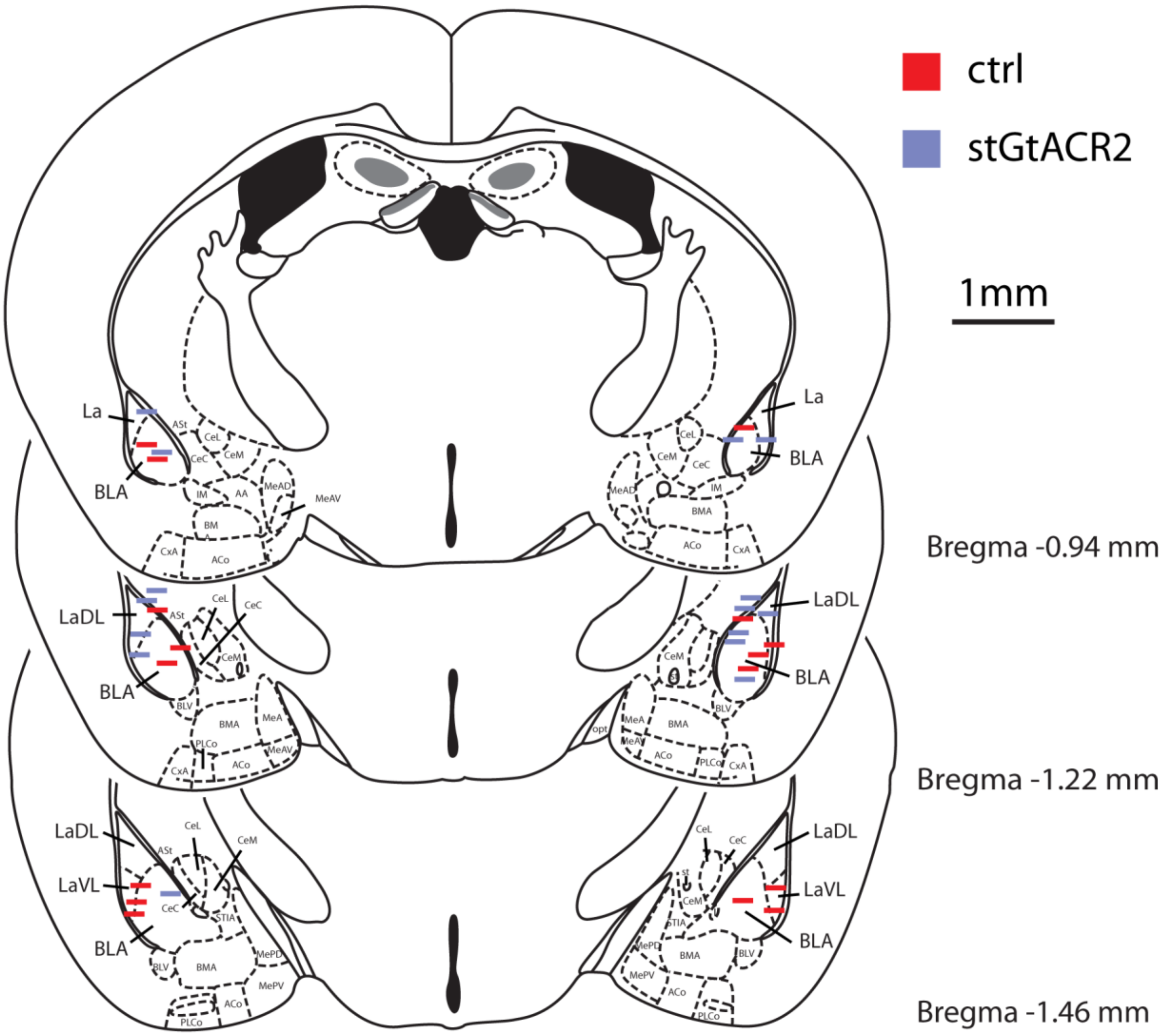

Supplementary figure 6. Summary of optical fiber placement of BLA silencing

Fiber placement was determined from fixed brain sections obtained from mice that underwent the fear extinction learning experiment. stGtACR2 expression in the BLA was verified in all mice. Horizontal lines mark the approximate position of the fiber tip. Schematics adapted with permission from Franklin K. and Paxinos G. 2008[5]

condition		control		GtACR2		stGtACR2	
mouse		1	2	1	2	1	2
# units		49	11	18	82	62	36
% suppressed units	125 μW mm^{-2}	2.04	0	0	25.61	54.84	47.22
	250 μW mm^{-2}	4.08	0	0	35.37	59.68	63.89
	500 μW mm^{-2}	0	9.09	5.56	51.22	62.9	58.33
	1000 μW mm^{-2}	4.08	9.09	5.56	41.46	58.06	52.78
rel. firing rate: suppressed units	125 μW mm^{-2}	1.07	1.07	0.42	0.69	0.22	0.18
	250 μW mm^{-2}	1.08	0.46	0.1	0.28	0.05	0.03
	500 μW mm^{-2}	1.07	1.6	0.08	0.11	0.05	0.01
	1000 μW mm^{-2}	0.74	0.18	0.02	0.07	0.05	0.02

Supplementary Table 1. Targeting GtACR2 to the somatodendritic compartment increases its efficacy of silencing *in vivo*.

Data represented in fig. 5 is listed per mouse. Relative firing rate values shown (bottom 4 rows) are the mean across all suppressed units.

Supplementary References

1. Yona, G., Meitav, N., Kahn, I. & Shoham, S., Realistic numerical and analytical modeling of light scattering in brain tissue for optogenetic applications. *eneuro* **3**, ENEURO--0059 (2016).

2. Liu, Y. *et al.*, OptogenSIM: a 3D Monte Carlo simulation platform for light delivery design in optogenetics. *Biomedical optics express* **6** (12), 4859-4870 (2015).

3. Chugh, B. P. *et al.*, Measurement of cerebral blood volume in mouse brain regions using micro-computed tomography. *NeuroImage* **47** (4), 1312-1318 (2009).

4. Govorunova, E. G., Sineshchekov, O. A., Janz, R., Liu, X. & Spudich, J. L., Natural light-gated anion channels: A family of microbial rhodopsins for advanced optogenetics. *Science* **349**, 647-650 (2015).

5. Franklin, K. B. & Paxinos, G., *The mouse brain in stereotaxic coordinates, compact. The coronal plates and diagrams* (Amsterdam: Elsevier Academic Press, 2008).
